# Social disadvantage, linguistic distance, ethnic minority status and first-episode psychosis: results from the EU-GEI case–control study

**DOI:** 10.1017/S003329172000029X

**Published:** 2021-07

**Authors:** Hannah E. Jongsma, Charlotte Gayer-Anderson, Ilaria Tarricone, Eva Velthorst, Els van der Ven, Diego Quattrone, Marta di Forti, EU-GEI WP2 Group, Paulo Rossi Menezes, Christina Marta Del-Ben, Celso Arango, Antonio Lasalvia, Domenico Berardi, Caterina La Cascia, Julio Bobes, Miguel Bernardo, Julio Sanjuán, Jose Luis Santos, Manuel Arrojo, Lieuwe de Haan, Andrea Tortelli, Andrei Szöke, Robin M. Murray, Bart P. Rutten, Jim van Os, Craig Morgan, Peter B. Jones, James B. Kirkbride

**Affiliations:** 1 PsyLife Group, Division of Psychiatry, UCL, London, England; 2 Department of Psychiatry, University of Cambridge, Cambridge, England; 3 Department of Health Services and Population Research, Institute of Psychiatry, Psychology and Neuroscience, King's College London, London, England; 4 Transcultural Psychosomatic Team (BoTPT), Department of Surgical and Medical Sciences, Bologna University, Bologna, Italy; 5 Department of Psychiatry, Icahn School of Medicine at Mount Sinai, New York, New York, USA; 6 Department of Preventative Medicine, Icahn School of Medicine at Mount Sinai, New York, New York, USA; 7 Early Psychosis Section, Department of Psychiatry, Amsterdam UMC, location AMC, Amsterdam, The Netherlands; 8 Department of Epidemiology, Mailman School of Public Health, Columbia University, New York, New York, USA; 9 Rivierduinen Institute for Mental Health Care, Leiden, The Netherlands; 10 Department of Psychiatry and Neuropsychology, School for Mental Health and Neuroscience, Maastricht University Medical Centre, Maastricht, The Netherlands; 11 Department of Psychosis Studies, Institute of Psychiatry, Psychology and Neuroscience, King's College London, London, England; 12 Department of Preventive Medicine, Faculdade de Medicina, University of São Paulo, São Paulo, Brazil; 13 Division of Psychiatry, Department of Neuroscience and Behaviour, Ribeirão Preto Medical School, University of São Paulo, São Paulo, Brazil; 14 Department of Child and Adolescent Psychiatry, Hospital General Universitario Gregorio Marañón, School of Medicine, Universidad Complutense, Investigación Sanitaria del Hospital Gregorio Marañón, Madrid, Spain; 15 Centro de Investigación Biomédica en Red de Salud Mental, Madrid, Spain; 16 Section of Psychiatry, Azienda Ospedaliera Universitaria Integra di Verona, Verona, Italy; 17 Department of Biomedical and Neuro-motor Sciences, Psychiatry Unit, Alma Mater Studiorum Università di Bologna, 40126 Bologna, Italy; 18 Unit of Psychiatry, ‘P. Giaccone’ General Hospital, Palermo, Italy; 19 Department of Medicine, Psychiatry Area, School of Medicine, Universidad de Oviedo, Centro de Investigación Biomédica en Red de Salud Mental, Instituto Investigación Sanitaria del Principado de Asturias, Oviedo, Spain; 20 Barcelona Clinic Schizophrenia Unit, Department of Medicine, Neuroscience Institute, Hospital Clinic, University of Barcelona, Institut d'Investigacions Biomèdiques August Pi i Sunyer, Centro de Investigación Biomédica en Red de Salud Mental, Barcelona, Spain; 21 Department of Psychiatry, School of Medicine, Universidad de Valencia, Centro de Investigación Biomédica en Red de Salud Mental, Valencia, Spain; 22 Department of Psychiatry, Servicio de Psiquiatría Hospital ‘Virgen de la Luz’, Cuenca, Spain; 23 Department of Psychiatry, Psychiatric Genetic Group, Instituto de Investigación Sanitaria de Santiago de Compostela, Complejo Hospitalario Universitario de Santiago de Compostela, Santiago de Compostela, Spain; 24 Etablissement Public de Santé Maison Blanche, Paris, France; 25 Institut National de la Santé et de la Recherche Médicale, U955, Créteil, France; 26 Department of Psychiatry, Brain Center Rudolf Magnus, Utrecht University Medical Centre, Utrecht, The Netherlands; 27 CAMEO Early Intervention Service, Cambridgeshire and Peterborough National Health Service Foundation Trust, Cambridge, England

**Keywords:** Discrimination, epidemiology, ethnicity, psychotic disorders, social disadvantage

## Abstract

**Background:**

Ethnic minority groups in Western countries face an increased risk of psychotic disorders. Causes of this long-standing public health inequality remain poorly understood. We investigated whether social disadvantage, linguistic distance and discrimination contributed to these patterns.

**Methods:**

We used case–control data from the EUropean network of national schizophrenia networks studying Gene-Environment Interactions (EU-GEI) study, carried out in 16 centres in six countries. We recruited 1130 cases and 1497 population-based controls. Our main outcome measure was first-episode ICD-10 psychotic disorder (F20–F33), and exposures were ethnicity (white majority, black, mixed, Asian, North-African, white minority and other), generational status, social disadvantage, linguistic distance and discrimination. Age, sex, paternal age, cannabis use, childhood trauma and parental history of psychosis were included as *a priori* confounders. Exposures and confounders were added sequentially to multivariable logistic models, following multiple imputation for missing data.

**Results:**

Participants from any ethnic minority background had crude excess odds of psychosis [odds ratio (OR) 2.03, 95% confidence interval (CI) 1.69–2.43], which remained after adjustment for confounders (OR 1.61, 95% CI 1.31–1.98). This was progressively attenuated following further adjustment for social disadvantage (OR 1.52, 95% CI 1.22–1.89) and linguistic distance (OR 1.22, 95% CI 0.95–1.57), a pattern mirrored in several specific ethnic groups. Linguistic distance and social disadvantage had stronger effects for first- and later-generation groups, respectively.

**Conclusion:**

Social disadvantage and linguistic distance, two potential markers of sociocultural exclusion, were associated with increased odds of psychotic disorder, and adjusting for these led to equivocal risk between several ethnic minority groups and the white majority.

## Introduction

People diagnosed with a psychotic disorder have a decreased life expectancy of 15 years compared with the general population (Hayes, Marston, Walters, King, & Osborn, 2017; Hjorthøj, Stürup, McGrath, & Nordentoft, 2017). The risk of developing such disorders inequitably affects migrants and their descendants in high-income countries (Selten, van der Ven, & Termorshuizen, 2020), making this a pressing public health concern, particularly in the context of accelerating global migration (Department of Economic and Social Affairs, 2017). Despite almost a century of research (Ødegaard, 1932), the causes of increased rates of psychotic disorder in ethnic minority groups remain poorly understood. They are not an artefact of demography: differences persist after adjusting for age, sex (Jongsma et al., 2018) and socioeconomic status (SES) (Kirkbride, Hameed, Ankireddypalli, et al., 2017). Whilst disparities in pathways to care (Anderson, Flora, Archie, Morgan, & McKenzie, 2014) and outcomes (Morgan et al., 2017) exist by ethnicity, there is little evidence that this explains differential rates between ethnic groups. Over-diagnosis in ethnic minority groups is not supported by evidence from clinical practice (Hickling, McKenzie, Mullen, & R, 1999; Lewis, Croft-Jeffreys, & David, 1990), nor by epidemiological studies using culturally-sensitive diagnostic instruments (Zandi et al., 2010) or partially-blinded, consensus-based research criteria to ascertain diagnoses (Fearon et al., 2006). Excess rates are also not ‘inherent’ to any ethnic group; for instance, incidence in people of black Caribbean heritage in the UK is up to five times higher than for the white British population (Kirkbride et al., 2006), a pattern not observed in estimates of incidence rates in Caribbean countries (Bhugra et al., 1996; Hickling, 1995). Whilst pre-migratory circumstances, including adversities experienced by refugees, may exacerbate risk (Brandt et al., 2019), post-migratory factors are also implicated, given the persistence of elevated risk in second-generation migrants (Selten et al., 2019). Given this persistence of excess risk in migrants and their descendants, we focus our investigation on the social context in high-income countries and use the term ethnic minorities throughout.

The social gradient in health, where those who are worse off socio-economically have worse health, might provide an explanation for this excess psychosis risk in some ethnic minority groups (Fisher & Baum, 2010; Marmot, 2006; Marmot et al., 2010). Social gradients are strongly patterned by ethnic minority status (Savage et al., 2013; Statistics Netherlands, 2018). Seen through this lens, health disparities arise via a process of (psychosocial) disempowerment, which is defined as experiencing a lack of control over one's life (Marmot, 2015). Individuals without sufficient social, economic, political or cultural capital required to achieve autonomy and control over their environment are exposed to more risk factors for poor health outcomes including psychotic disorders. Risk factors include lower education, SES and social isolation (Marmot, 2006; Marmot et al., 2010; Public Health England & UCL Institute of Health Equity, 2017).

Here, we propose that excess risks of psychotic disorder in several ethnic minority groups may arise through such a process of psychosocial disempowerment, following greater exposure to social disadvantage (Savage et al., 2013; Statistics Netherlands, 2018) and exclusion based on cultural and ethnic identity (Akerlof & Kranton, 2011; Nazroo & Karlsen, 2003; Smaje, 1996), including language barriers and overt experiences of discrimination. To test this hypothesis empirically, we examined whether individual-level social disadvantage (an indication of the ethnic patterning of the social gradient in health), linguistic distance and experiences of discrimination differed between ethnic groups, and tested whether this accounted for differences in the risk of psychotic disorders by ethnicity and generational status. We used data from the six-country EUropean network of national schizophrenia networks studying Gene-Environment Interactions (EU-GEI; work package 2) case–control study, which included these exposure measures in an ethnically- and culturally-diverse sample.

## Methods

### Study design and participants

Participants were recruited in 17 centres in England, the Netherlands, Spain, France, Italy and Brazil, between 2010 and 2015 (Jongsma et al., 2018). All persons aged 18–64 years who made contact with mental health services for a probable first-episode of psychosis (FEP) were invited to participate via their mental healthcare provider. Cases were included if they subsequently met International Classification of Disease (ICD)-10 criteria for a psychotic disorder (F20–33), ascertained using the Operational Criteria Checklist (OPCRIT) algorithm [detailed fully elsewhere (Jongsma et al., 2018)]. We included non-affective psychotic disorders (ICD-10 codes F20–25) and affective psychotic disorders (ICD-10 codes F30–F33) as secondary outcomes.

In each centre, we recruited controls from the population-at-risk (individuals who never had an FEP). We used random sampling methods (e.g. via general practice lists in the UK) and set quotas to ensure that our control sample was representative of the age–sex–ethnicity structure of the population-at-risk. Controls with a history of psychotic disorder, or taking anti-psychotic medication, were excluded. Some centres purposively over-sampled hard-to-reach groups to increase representativeness (online Supplementary Methods).

We excluded participants with insufficient exposure data to estimate linguistic distance, and cases for whom an OPCRIT diagnosis could not be completed. The authors assert that all procedures contributing to this work comply with the ethical standards of the relevant national and institutional committees on human experimentation and with the Helsinki Declaration of 1975, as revised in 2008. All procedures involving participants were approved by the following respective local ethics committees: South London and Maudsley and Institute of Psychiatry Research Ethics Committee; National Research Ethics Service Committee East of England–East Cambridge; Medisch-Ethische Toetsingscommissie van het Academisch Centrum te Amsterdam; Comité Ético de Investigación Clínica Hospital Gregorio Marañón; Comité Ético de Investigación Clínica del Hospital Clinic de Barcelona; Comité Ético de Investigación Clínica del Hospital Clinic Universitari de Valencia; Comité Ética de la Investigación Clínica del Principado de Asturias; Comité Ético de Investigación Clínica de Galicia; Comité Ético de Investigación Clínica del Hospital Virgen de la Luz de Cuenca; Comité de Protéction des Personnes–CPP Île de France IX; Comitato Etico Policlinico S Orsola Malpighi; Comitato Etico Azienda Ospedaleria Universitaria di Verona; Comitato Etico Palermo 1, Azienda Ospedaliera Policlinico ‘Paolo Giaccone’; and Research Ethics Committee of the clinical Hospital of Ribeirão Preto Medical School, University of São Paulo, Brazil. All participants gave written informed consent (Di Forti et al., 2019; Jongsma et al., 2018).

### Measures

Our main exposures were indicator variables which operationalised the constructs of ethnicity, social disadvantage, linguistic distance and self-perceived discrimination, obtained from an amended version of the Medical Research Council Socioeconomic Schedule (MRC SDS) (Di Forti et al., 2019; Mallett, 1997). Ethnic group was coded by self-ascription to seven categories: white majority (reference category, i.e. in English sites, white British), black, mixed, Asian, north African, white minority and other (see online Supplementary Methods). We chose the white majority as our reference category, as in each country, this referred to the majority population. We also examined results by generational status (first- or later-generation), based on place of birth and ethnicity. We defined a set of indicators of social disadvantage (including social functioning) to include educational attainment (no qualifications; school qualifications; tertiary; vocational; undergraduate; postgraduate), lifetime relationship status [ever/never in a long-term (1 < year) relationship], lifetime living arrangements (lived with people other than parents; yes/no) and parental SES. This was based on the main breadwinner's highest occupation, categorised from the European Socio-economic Classification (Harrison & Rose, 2006) to six categories: professional (higher and lower grade), intermediate (intermediate occupations, small employers, self-employed), lower (supervisory, technician, services, sales, clerical and technical), routine, never worked (including long-term unemployed) and not classified (including students).

Linguistic distance was operationalised using two measures: language distance and fluency in the majority language (Candelo, Croson, & Li, 2017; Koczan, 2016; West & Graham, 2004). We estimated language distance by scoring each participant's first language as a function of distance on a language tree from the majority language in their country of residence (i.e. England, France, Spain, etc). Scores were rated from 0 (participant first language same as majority language in the country of residence) to 3 (participant first language from a different language family to majority language; see online Supplementary Methods and Fig. S1). The face validity of this approach was confirmed by an expert in linguistics (JvdW). Fluency in the majority language was a single, self-rated item and was rated on a 10-point scale. Due to skew on both measures (online Supplementary Figs S2 and S3), we created a binary linguistic distance variable: no linguistic distance (language distance = 0, fluency = 10) or some linguistic distance (language distance ⩾1 and/or fluency ⩽9). We measured all-cause self-perceived discrimination continuously, using a 12-item version of the Major Experiences of Discrimination questionnaire (Williams, Yu, Jackson, & Anderson, 1997) (online Supplementary Methods).

We also adjusted for sex, parental history of psychosis, lifetime cannabis use (all binary), age, paternal age and childhood trauma (all continuous) as *a priori* confounders. Age, sex and paternal age were derived from the MRC SDS. Parental history of psychosis was recorded using the Family Interview for Genetic Studies questionnaire (NIHM Center for Collaborative Genomics Research on Mental Disorders, 2017). Childhood trauma was operationalised as the total score on the Childhood Trauma Questionnaire (Bernstein et al., 2003), and cannabis use was derived from the Cannabis Experience Questionnaire (Barkus, Stirling, Hopkins, & Lewis, 2006).

### Missing data

Missing data were handled via multiple imputation (MI) by fully-conditional specification using chained equations (Little & Rubin, 2002; Sterne et al., 2009). Analyses were conducted post-imputation, combining estimates across 25 imputed data sets using Rubin's rule (White, Royston, & Wood, 2011). We included all covariates and several auxiliary variables in our MI algorithm (online Supplementary Methods).

### Statistical analyses

We first presented descriptive statistics using χ^2^ tests and Mann–Whitney *U* tests (MWU), including investigating patterns of missingness by case–control status. We used polychoric correlations to describe associations between confounders and exposures in the control sample. We used multinomial regression to examine associations between ethnicity and other covariates. Following MI, we fitted sequential multilevel logistic regression models, with random intercepts at the centre level to account for the hierarchical nature of the dataset (individuals within centres), to investigate the association between ethnicity and case–control status, as follows:

• Crude (univariable) association between case–control status and ethnicity
• Model A: adjusted for *a priori* confounders (age, sex, paternal age, parental history of psychosis, cannabis use and childhood trauma)
• Model B: Model A + social disadvantage (parental SES, education level, relationship status, living arrangements)
• Model C: Model B + linguistic distance
• Model D: Model C + self-perceived discrimination.

We re-ran our models substituting ethnicity for first- *v.* later-generation migrant status, and using our secondary outcomes. We performed sensitivity analyses on complete cases only for the primary outcome (using inverse probability weights to account for the sampling design, see online Supplementary Methods). We presented odds ratios (OR) and 95% confidence intervals (95% CI) where appropriate, and analysed data using Stata 14 (StataCorp, 2015).

The strengthening the reporting of observational studies in epidemiology (STROBE) checklist (von Elm et al., 2007) and the original analysis plan, approved internally by the EU-GEI team in August 2016, are included in online Supplementary Tables S2 and S3.

## Results

We recruited 1130 cases and 1497 controls into the study (Di Forti et al., 2019). Following exclusion of participants with missing linguistic distance (*N* = 2 cases, 0.2%; *N* = 2 controls, 0.1%), cases from our Paris centre (where no controls were recruited, *N* = 36, 3.1%) and cases without OPCRIT (*n* = 4, 0.4%), the final sample size was 2583 (*N* = 1088 cases, *N* = 1495 controls; 98.3% of total recruited). A total of 761 (70.0%) cases presented with a non-affective psychotic disorder, and 306 (28.1%) with an affective psychotic disorder. A further 21 (1.9%) cases presented with psychosis not otherwise specified and were not included for secondary outcome analysis. Controls were broadly representative of the population-at-risk on sex and ethnic minority status, but were younger than the population-at-risk (online Supplementary Results and Supplementary Table S4).

### Missing data

The proportion of missing covariate data was generally low (Table 1), ranging from two participants (0.1%) on age, to 301 (11.7%) on parental history of psychosis. Cases were more likely to be missing data on paternal age [*n* = 140 (12.9%) *v. n* = 55 (3.6%)], cannabis use [*n* = 29 (2.7%) *v. n* = 16 (1.1%)], childhood trauma [*n* = 89 (8.2%) *v. n* = 12 (0.8%)], relationship status [*n* = 10 (0.9%) *v. n* = 1 (0.1%)] and self-perceived discrimination [*n* = 87 (8.0%) *v. n* = 66 (4.4%)], but not on other covariates (Table 1).

**Table 1.** Distribution of exposures and covariates by case-control status.

Variable		Controls *n* (*%*)	Cases *n* (*%*)	χ^2^; *p*-value/MWU^a^; *p*-value
Age	Median (IQR)	33 (26–47)	29 (22–37)	MWU: 9.28, *p* < 0.0001
	Missing	2 (0.1%)	–	
Sex	Men	705 (47.2%)	671 (61.7%)	χ^2^: 53.30, *p* < 0.0001
	Women	790 (52.8%)	417 (38.3%)	
	Missing	–	–	
Paternal age	Median (IQR)	31 (27–35)	31 (27–36)	MWU: −0.59, *p* = 0.56
	Missing (%)	55 (3.6%)	140 (12.9%)	
Childhood trauma	Median (IQR)	31 (27–38)	39 (32–49)	MWU: −16.70, *p* < 0.0001
	Missing (%)	12 (0.8%)	89 (8.2%)	
Cannabis use	Yes	702(46.9%)	683 (62.8%)	χ^2^: 79.76, *p* < 0.0001
	No	777 (51.9%)	376 (34.6%)	
	Missing	16 (1.1%)	29 (2.7%)	
Parental history of psychosis	Yes	23 (1.5%)	68 (6.3%)	χ^2^: 43.67, *p* < 0.0001
	No	1307 (87.4%)	884 (81.3%)	
	Missing	165 (11.0%)	136 (12.6%)	
Generational status	Not applicable (majority)	1081 (72.3%)	639 (58.2%)	χ^2^: 53.87, *p* < 0.0001
	First generation	218 (15.6%)	238 (21.8%)	
	Second generation	196 (13.1%)	215 (19.7%)	
	Missing	–	–	
Ethnicity	White majority	1084 (72.4%)	634 (58.3%)	χ^2^: 70.99, *p* < 0.0001
	Black	132 (8.3%)	168 (15.4%)	
	Mixed	96 (6.4%)	107 (9.8%)	
	Asian	33 (2.2%)	33 (3.1%)	
	North African	25 (1.7%)	45 (4.1%)	
	Other	25 (1.7%	29 (2.8%)	
	White other^b^	100 (6.7%)	72 (6.8%)	
	Missing	–	–	
Parental SES	Professional	440 (29.4%)	242 (22.2%)	χ^2^: 45.16, *p* < 0.0001
	Intermediate	315 (21.1%)	209 (19.2%)	
	Lower	386 (25.8%)	287 (26.4%)	
	Routine	2226 (15.1%)	186 (17.1%)	
	Never worked	3 (0.2%)	15 (1.4%)	
	Not classified	60 (4.0%)	64 (2.9%)	
	Missing	65 (4.4%)	85 (7.8%)	
Level of education	Postgraduate	209 (14.0%)	52 (4.8%)	χ^2^: 251.45, *p* < 0.0001
	Undergraduate	343 (22.9%)	122 (11.2%)	
	Vocational	236 (15.8%)	192 (17.6%)	
	Tertiary	431 (28.8%)	254 (23.3%)	
	School qualifications	197 (13.2%)	280 (25.7%)	
	School, no qualifications	72 (4.8%)	177 (16.3%)	
	Missing	7 (0.5%)	11 (1.0%)	
Relationship status	Yes	1333 (89.0%)	733 (67.4%)	χ^2^: 189.09, *p* < 0.0001
	No	161 (10.8%)	345 (31.7%)	
	Missing	1 (0.2%)	10 (0.9%)	
Living arrangements	Yes	1218 (81.5%)	742 (68.1%)	χ^2^: 60.70, *p* < 0.0001
	No	257 (17.2%)	323 (29.7%)	
	Missing	20 (1.3%)	23 (2.0%)	
Linguistic distance	Yes	144 (9.6%)	186 (17.1%)	χ^2^: 31.48, *p* < 0.0001
	No	1351 (90.4%)	902 (82.9%)	
	Missing	–	–	
Discrimination	Median (IQR)	0 (0–1)	0 (0–2)	MWU: −4.76, *p* < 0.0001
	Missing	66 (4.4%)	87 (8.0%)	

a MWU: Mann–Whitney *U* test used to test for differences in median value between cases and controls.

b White other refers to white minority participants (see Supplemental Table 1).

### Demographic characteristics

Cases were more likely than controls to be black, mixed, North African or of ‘other’ ethnicity (χ^2^ 71.0, *p* < 0.01; Table 1). Controls had higher education (χ^2^ 251.5, *p* < 0.01), were more likely to have ever been in a relationship (χ^2^ 189.1, *p* < 0.01) and to have lived with someone other than their parents (χ^2^ 60.7, *p* < 0.01). Cases reported greater linguistic distance (χ^2^ 31.5, *p* < 0.01) and discrimination (MWU −4.8, *p* < 0.01), were younger (MWU 9.3, *p* < 0.01), more likely to be male (χ^2^ 53.3, *p* < 0.01), to have smoked cannabis (χ^2^ 79.8, *p* < 0.01), to have a parental history of psychosis (χ^2^ 43.7, *p* < 0.01), to have experienced childhood trauma (MWU −16.7, *p* < 0.01) and to have lower parental SES (χ^2^ 45.2, *p* < 0.01) than controls. We found no difference in paternal age (MWU −0.6, *p* = 0.56). Correlations between exposures and confounders in the control sample were generally very weak, and are detailed in online Supplementary Table S5.

Multinomial regression using the white majority as the reference category revealed that all ethnic minority groups reported greater linguistic distance and discrimination than the white majority (Table 2), being highest in North African (OR_linguistic distance_ 5.41, 95% CI 4.72–6.10) and ‘other’ ethnic minority groups (OR_discrimination_ 1.59, 95% CI 1.40–1.82). The distribution of linguistic distance by ethnic group and generational status can be found in online Supplementary Figs 4 and 5. Findings for social disadvantage were more mixed. Participants from black, mixed and North African ethnic backgrounds had lower parental SES than the white majority, in contrast to participants from Asian, white minority and ‘other’ ethnicities. Similar patterns were apparent for education and relationship status (Table 2). While most ethnic minority groups reported more childhood trauma than the white majority, there was no evidence of higher lifetime cannabis use in any ethnic minority group; further, only participants of black ethnicity reported a greater parental history of psychosis (Table 2).

**Table 2.** Multinomial regression of ethnicity on other covariates

	Ethnic minority group					
	Black	Mixed	Asian	North African	Other	White minority
Variable	OR (95% CI)	OR (95% CI)	OR (95% CI)	OR (95% CI)	OR (95% CI)	OR (95% CI)
Discrimination (0–12)	**1.51** (**1.40–1.62)**	**1.18** (**1.06–1.32)**	**1.28** (**1.09–1.51)**	**1.50** (**1.32–1.71)**	**1.59** (**1.40–1.82)**	**1.27** (**1.14–1.42)**
Linguistic distance						
No	Reference	Reference	Reference	Reference	Reference	Reference
Yes	**2.45 (2.92–3.97)**	1.64 (0.87–2.39)	**4.86 (4.19–5.53)**	**5.41 (4.72–6.10)**	**4.61 (3.95–5.33)**	**4.96 (4.41–5.51)**
Parental SES						
Professional	Reference	Reference	Reference	Reference	Reference	Reference
Intermediate	0.97 (0.67–1.41)	1.21 (0.71–2.07)	0.81 (0.45–1.46)	**2.55 (1.08–6.03)**	1.13 (0.54–2.34)	0.81 (0.53–1.27)
Lower	0.87 (0.62–1.24)	**1.92 (1.21–3.05)**	**0.22 (0.09–0.56)**	**2.66 (1.18–604)**	0.73 (0.34–1.56)	0.71 (0.47–1.08)
Routine	**1.48 (1.02–2.15)**	**4.42 (2.78–7.02)**	0.51 (0.22–1.20)	**4.64 (2.00–10.76)**	1.09 (0.47–2.49)	0.68 (0.40–1.15)
Never worked	1.86 (0.53–7.26)	**5.22 (1.35–20.19)**	n/a	**11.63 (2.17–62.46)**	n/a	0.90 (0.11–7.09)
Not classified	1.20 (0.65–2.16)	**2.50 (1.26–5.00)**	n/a	0.69 (0.09–5.51)	0.68 (0.15–3.05)	0.69 (0.30–1.57)
Level of education						
Postgraduate	Reference	Reference	Reference	Reference	Reference	Reference
Undergraduate	**1.90 (1.01–3.61)**	1.48 (0.61–3.57)	0.51 (0.24–1.10)	0.80 (0.22–2.88)	0.46 (0.18–1.16)	**0.67 (0.22–0.62)**
Vocational	**2.74 (1.46–5.15)**	1.95 (0.82–1.65)	0.44 (0.19–1.01)	2.80 (0.93–8.40)	0.80 (0.34–1.85)	**0.36 (0.20–0.62)**
Tertiary	**2.09 (1.13–3.86)**	**2.69 (1.20–6.04)**	**0.34 (0.17–0.73)**	1.40 (0.46–4.25)	**0.33 (0.13–0.82)**	**0.41 (0.26–0.66)**
School qualifications	**2.43 (1.29–4.57)**	**4.51 (2.01–10.13)**	0.49 (0.22–1.08)	2.30 (0.76–6.99)	0.57 (0.23–1.40)	**0.39 (0.23–0.67)**
No qualifications	**5.43 (2.82–40.42)**	**9.41 (4.11–21.52)**	0.30 (0.08–1.08)	**3.55 (1.09–11.56)**	0.71 (0.24–2.12)	**0.45 (0.22–0.89)**
Relationship status						
No	Reference	Reference	Reference	Reference	Reference	Reference
Yes	**0.67 (0.49–0.89)**	**0.67 (0.48–0.95)**	0.76 (0.42–1.36)	**0.45 (0.27–0.76)**	0.74 (0.39–1.38)	**1.64 (1.01–2.67)**
Living arrangements						
No	Reference	Reference	Reference	Reference	Reference	Reference
Yes	1.30 (0.96–1.75)	**1.51 (1.04–2.19)**	1.14 (0.63–2.05)	1.23 (0.68–2.19)	2.07 (0.97–4.41)	**3.50 (2.03–6.01)**
Age	**0.97 (0.96–0.98)**	**0.97 (0.95–0.98)**	**0.97 (0.95–0.99)**	0.99 (0.97–1.01)	0.98 (0.95–1.00)	1.00 (0.99–1.01)
Sex						
Female	Reference	Reference	Reference	Reference	Reference	Reference
Male	1.07 (0.83–1.36)	0.85 (0.63–1.14)	1.14 (0.70–1.87)	**1.95 (1.17–3.26)**	1.19 (0.70 −2.04)	0.97 (0.71–1.33)
Paternal age	1.00 (0.99–1.02)	0.99 (0.97–1.01)	1.00 (0.97–1.04)	**1.06 (1.03–1.09)**	1.01 (0.97–1.05)	1.00 (0.97–1.02)
Childhood trauma	**1.04 (1.03–1.05)**	**1.04 (1.03–1.05)**	1.02 (1.00–1.04)	**1.03 (1.02–1.05)**	**1.04 (1.02–1.06)**	**1.02 (1.01–1.03)**
Cannabis use						
No	Reference	Reference	Reference	Reference	Reference	Reference
Yes	0.89 (0.69–1.13)	**0.64 (0.48–0.85)**	0.87 (0.57–1.43)	1.10 (0.38–1.79)	1.27 (0.73–2.20)	1.25 (0.90–1.73)
Parental psychosis						
No	Reference	Reference	Reference	Reference	Reference	Reference
Yes	**1.85 (1.07–3.19)**	1.18 (0.58–2.42)	–	0.40 (0.05–2.87)	0.73 (0.11–4.72)	1.18 (0.51–2.77)

Odds ratios are relative to the White majority group.

Odds ratios in **bold** are statistically significant (*p* < 0.05).

### Multivariable modelling

In unadjusted models, ethnic minority status was associated with increased odds of psychotic disorders (OR 2.03, 95% CI 1.69–2.43), being highest for North African (OR 3.72, 95% CI 2.18–6.34) and black participants (OR 2.49, 95% CI 1.88–3.28) (Table 3). Greater social disadvantage, linguistic distance (OR 1.94, 95% CI 1.52–2.48) and self-perceived discrimination (OR per unit increase: 1.20, 95% CI 1.12–1.27) also showed strong univariable associations with psychosis risk (Table 3).

**Table 3.** Odds of psychotic disorders by exposure status following incremental covariate adjustment

Model	Crude	Model A^a^	Model B^b^	Model C^c^	Model D^d^
Variable	OR (95% CI)	OR (95% CI)	OR (95% CI)	OR (95% CI)	OR (95% CI)
Ethnicity					
White native	Reference	Reference	Reference	Reference	Reference
All minorities	**2.03** (**1.69–2.43)**	**1.62** (**1.32–1.98)**	**1.54** (**1.24–1.92)**	1.23 (0.96–1.59)	1.21 (0.94–1.56)
Black	**2.49 (1.88–3.28)**	**1.85 (1.36–2.52)**	**1.53 (1.10–2.13)**	1.32 (0.94–1.85)	1.29 (0.92–1.82)
Mixed	**2.29 (1.67–3.15)**	**1.74 (1.23–2.45)**	1.41 (0.97–2.05)	1.34 (0.92–1.96)	1.32 (0.91–1.93)
Asian	**1.74 (1.05–2.88)**	1.64 (0.95–2.81)	**2.03 (1.13–3.65)**	1.44 (0.77–2.68)	1.42 (0.76–2.65)
North-African	**3.72 (2.18–6.34)**	**3.12 (1.73–5.61)**	**2.58 (1.38–4.84)**	1.69 (0.86–3.33)	1.65 (0.84–3.26)
Other	**1.88 (1.08–3.30)**	1.34 (0.73–2.46)	1.51 (0.79–2.89)	1.10 (0.56–2.18)	1.06 (0.54–2.11)
White other	1.24 (0.89–1.72)	1.13 (0.79–1.62)	1.35 (0.92–1.99)	0.93 (0.60–1.44)	0.93 (0.60–1.44)
Parental SES		n/a			
Professional	Reference		Reference	Reference	Reference
Intermediate	1.24 (1.00–1.62)		1.07 (0.81–1.41)	1.08 (0.82–1.43)	1.08 (0.82–1.43)
Lower	**1.49 (1.18–1.88)**		1.11 (0.84–1.46)	1.12 (0.85–1.48)	1.12 (0.85–1.48)
Routine	**1.74 (1.33–2.27)**		0.94 (0.69–1.29)	0.97 (0.71–1.34)	0.97 (0.71–1.34)
Never worked	**8.37 (2.38–29.43)**		3.20 (0.83–12.39)	3.31 (0.84–13.14)	3.33 (0.84–13.26)
Not classified	**2.35 (1.57–3.53)**		**2.10 (1.32–3.34)**	**2.11 (1.33–3.36)**	**2.11 (1.33–3.36)**
Level of education		n/a			
Postgraduate	Reference		Reference	Reference	Reference
Undergraduate	**1.59 (1.05–2.23)**		1.42 (0.95–2.13)	1.42 (0.95–2.1)	1.43 (0.95–2.14)
Vocational	**3.49 (2.42–5.06)**		**2.96 (1.73–3.89)**	**2.59 (1.73–3.89)**	**2.57 (1.71–3.86)**
Tertiary	**2.92 (2.04–4.18)**		**1.86 (1.26–2.75)**	**1.86 (1.26–2.76)**	**1.86 (1.26–2.75)**
School qualifications	**7.05 (4.87–10.20)**		**4.77 (3.18–7.15)**	**4.81 (3.20–7.22)**	**4.81 (3.20–7.22)**
No qualifications	**13.04 (8.46–20.09)**		**8.24 (5.07–13.37)**	**8.15 (5.02–13.25)**	**8.17 (5.03–13.28)**
Relationship status		n/a			
No	Reference		Reference	Reference	Reference
Yes	**0.26 (0.21–0.32)**		**0.34 (0.27–0.44)**	**0.34 (0.26–0.44)**	**0.34 (0.26–0.44)**
Living arrangements		n/a			
No	Reference		Reference	Reference	Reference
Yes	**0.47 (0.39–0.58)**		0.83 (0.64–1.06)	0.81 (0.62–1.04)	0.80 (0.62–1.03)
Linguistic distance		n/a	n/a		
No	Reference			Reference	Reference
Yes	**1.94 (1.52–2.48)**			**1.91 (1.33–2.76)**	**1.89 (1.31–2.73)**
Discrimination (0–12)	**1.20 (1.12–1.27)**	n/a	n/a	n/a	1.04 (0.97–1.12)

Odds ratios in **bold** are statistically significant (*p* < 0.05).

a Model A is adjusted for covariates (age, sex, their interaction, paternal age, childhood trauma, cannabis use, parental history of psychosis).

b Model B is further adjusted for indicators of social disadvantage (paternal SES, level of education, relationship status and living arrangements).

c Model C is further adjusted for linguistic distance.

d Model D is further adjusted for discrimination.

**Fig. 1.**
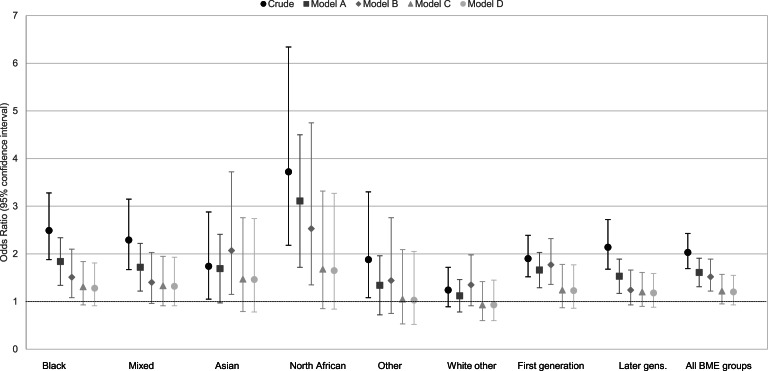
Odds of psychotic disorders, by ethnic group following incremental covariate adjustment. Model A: adjusted for covariates (age, sex, their interaction, paternal age, childhood trauma, cannabis use, parental history of psychosis). Model B: Model A+adjustment for indicators of social disadvantage (parental SES, level of education, relationship status and living arrangements). Model C: Model B+adjustment for linguistic distance. Model D: Model C+adjustment for discrimination.

Adjustment for *a priori* confounders (Model A, Table 3 and Fig. 1) led to some attenuation in psychosis risk in the overall ethnic minority group (OR 1.62, 95% CI 1.32–1.98), though excess odds remained for participants of North African (OR 3.12, 95% CI 1.73–5.61), black (OR 1.85, 95% CI 1.36–2.52) and mixed (OR 1.74, 95% CI 1.23–2.45) ethnicities, principally driven by childhood trauma (data available from authors). Adjustment for social disadvantage (Model B, Table 3) led to further attenuation in risk for all ethnic minority groups (OR 1.54, 95% CI 1.24–1.92), but negatively confounded the association between Asian ethnicity and psychosis to increase the risk (OR 2.03, 95% CI 1.13–3.65). Additional adjustment for linguistic distance (Model C, Table 3), which remained strongly associated with psychosis risk (OR 1.91, 95% CI 1.33–2.76), further attenuated psychosis risk in all ethnic minority groups (OR 1.23, 95% CI 0.96–1.59), such that for any specific ethnic minority group, we were unable to reject the null hypothesis. The addition of self-perceived discrimination did not alter ORs, and discrimination itself was no longer associated with psychosis risk in a multivariable model (Model D: OR 1.04, 95% CI 0.97–1.142).

### Risk in first- *v.* later-generation migrant groups

In crude models, first- and later-generation ethnic minority groups were at similarly elevated risk of psychotic disorder (online Supplementary Table S6), which persisted after adjustment for *a priori* confounders. Incremental adjustment for other covariates suggested that later-generation groups were no longer at increased odds of psychosis after adjustment for social disadvantage (Model B: OR 1.24; 95% CI 0.93–1.66), but the risk remained elevated for first-generation migrants (OR 1.82, 95% CI 1.39–2.38) until further adjustment for linguistic distance (Model C: OR 1.28, 95% CI 0.89–1.83; online Supplementary Table S6 and Fig. S1).

### Secondary outcomes

Secondary outcomes followed a similar pattern to our primary outcome, with crude excess odds observed in the overall ethnic minority group for both non-affective (OR 2.08, 95% CI 1.70–2.57; online Supplementary Table S7) and affective psychotic disorders (OR 1.84, 95% CI 1.40–2.42, online Supplementary Table S8). These associations were attenuated following adjustment for *a priori* confounders and social disadvantage, and were no longer increased following adjustment for linguistic distance (OR non-affective disorders: 1.17, 95% CI 0.87–1.58; OR affective disorders: 1.22, 95% CI 0.85–1.74). Due to the smaller sample sizes for each outcome, these estimates were accompanied by less precision, particularly for affective psychotic disorders.

### Sensitivity analyses

Model building (Models A–D) was repeated on the weighted complete-case sample for all psychotic disorders; results were similar to imputed analyses (online Supplementary Table S9).

## Discussion

We showed that a greater frequency of psychotic disorders typically observed in several ethnic minority groups (Selten et al., 2020) may be attributable to markers of social disadvantage and linguistic distance. The latter appeared to have stronger effects in first-generation migrants, while the former had greater magnitude amongst second- and later-generation ethnic minority groups. These findings were independent of several other putative confounders included in our models and were replicated across dichotomised diagnostic categories.

### Strengths and limitations

Our data were taken from a large, international case–control study with well-characterised socio-environmental exposures, using population-based control samples. Although missing data were generally low, we used MI to minimise the loss of precision or selection biases, which may have been introduced in complete-case analyses. We took a multilevel modelling approach to accurately estimate standard errors in nested data across sites, and an *a priori* modelling approach to reduce the plausibility of type I error.

Controls were broadly representative of the population-at-risk by sex and minority status, but were – on average – younger. Weighted complete-case sensitivity analyses, however, did not alter the interpretation of our results. We were unable to investigate representativeness for other covariates such as SES, as these were not available for the population-at-risk consistently across all six countries. We used the same validated instruments across settings, and standardised data-entry to minimise other forms of differential measurement bias. To minimise recall bias, we operationalised covariates broadly, or used well-validated measures. While we cannot exclude the possibility of differential recall between cases and controls, we have no reason to believe this would have differed by ethnicity. We acknowledge likely within-group heterogeneity inherent to our definition of ethnicity. Narrower definitions of ethnicity (e.g. black Caribbean) were unfeasible due to small sample sizes resulting from country-specific minority groups. Nevertheless, the consistency of our findings across each broad ethnic group increases the validity of our observations.

The association between linguistic distance and psychosis was novel, but needs considering in light of limitations, including the validity of this measure; linguistic distance showed apparent validity, being greater amongst all ethnic minority groups (with the exception of people from mixed ethnic backgrounds) than the majority population. Treating linguistic distance as a binary variable may have led to some residual confounding, but was necessary because of substantial skew in the underlying two items capturing linguistic distance. We also acknowledge that our measure may not have captured all aspects of cultural distance, including outsider status, and will not provide a complete account of the excess risk of psychotic disorders observed in second- and later-generation migrants (Bourque, van der Ven, & Malla, 2011; Selten et al., 2020), because one would expect little linguistic distance from the majority population. Indeed, our findings suggested that social disadvantage was a bigger driver of excess odds of psychosis in this group. Nonetheless, our results suggest further development and validation of measures which capture this construct is warranted. We also suggest that acculturative experiences, which are partly shaped by both social disadvantage and linguistic distance, should be studied in suitable longitudinal cohorts. Such designs would also minimise further limitations of this study, including the issue of non-collapsibility of ORs, a statistical property of ORs which might preclude interpreting them as risks. Nevertheless, given that the rare disease assumption is likely satisfied in our study, we do not believe non-collapsibility will have affected our results (Burgess, 2017; Vanderweele, 2016). Longitudinal research will also provide prospectively collected data to disentangle the potential role of reverse causality, particularly around social disadvantage and psychosis.

### Comparison with existing literature

Increased odds of psychotic disorders in ethnic minority groups are consistent with existing literature (Anderson, Cheng, Susser, McKenzie, & Kurdyak, 2015; Bourque et al., 2011; Kirkbride et al., 2012; Selten et al., 2020), particularly for people with black and mixed ethnic backgrounds (Kirkbride et al., 2012; Kirkbride, Hameed, Ioannidis, et al., 2017; Selten et al., 2019). Literature on the North African group is mixed, with strong evidence of increased incidence in Moroccan groups in the Netherlands (Veling et al., 2006), although not France (Tortelli et al., 2014). Our finding of excess odds of psychotic disorder in Asian groups in Europe supports some previous findings (Coid et al., 2008; Kirkbride et al., 2012; Kirkbride, Hameed, Ioannidis, et al., 2017), but, as for all ethnicities, will mask considerable heterogeneity within this group. No evidence of excess risk was found amongst white minority groups, in line with some (Bourque et al., 2011; Kirkbride, Hameed, Ioannidis, et al., 2017) but not all studies (Dykxhoorn et al., 2018). Such differences highlight the importance of investigating variability in minority group experiences in different contexts in future studies.

Previous studies have suggested that increased psychosis risk in ethnic minority groups is only partially attenuated by current SES (Kirkbride et al., 2008; Kirkbride, Hameed, Ioannidis, et al., 2017), consistent with our results. Here, social disadvantage had pernicious effects on psychosis risk, consistent with the previous work on this issue (Morgan et al., 2008), and our results are consistent with a socio-developmental model of psychosis in minorities (Morgan, Charalambides, Hutchinson, & Murray, 2010); our work suggests that psychosocial and cultural factors may be integral to such models. In our study, discrimination was strongly associated with the odds of psychotic disorder, and was more common amongst all ethnic minority groups. However, after adjusting for other markers of psychosocial disempowerment, no direct effect of discrimination remained. This partially accords with evidence from a previous study which found that whilst discrimination was associated with excess psychosis risk in ethnic minorities (Veling et al., 2007), it did not fully explain an account for it, while a further case–control study found no association (Veling, Hoek, & Mackenbach, 2008). Previous studies have reported that a greater proportion of people from one's own ethnic group in a given community attenuate psychosis risk for individuals from an ethnic minority background (Bécares, Nazroo, & Stafford, 2009). This so-called ‘ethnic density’ effect is thought to operate through social support garnered from one's own ethnocultural group (Bécares et al., 2009), but was not the focus of our study. However, our results suggest that in addition to such a mechanism, social and linguistic barriers which could limit people's capability to participate fully in the society may also be associated with psychosis risk. Neither mechanism needs to be mutually exclusive, and we require theoretical models which recognise the realistic complexity through which different contextual factors affect psychosis risk.

### Interpretation of findings

One possible explanation of our findings is that social disadvantage and linguistic distance increase psychosis risk in some ethnic minority groups via mechanisms such as psychosocial disempowerment (Marmot, 2015) or social defeat (Selten & Cantor-Graae, 2005). Multiple disadvantage is disproportionately concentrated in minority groups (Wilson, 2010) and in concert with cultural factors (Smaje, 1996) may act to disenfranchise and distance minority groups from the majority population who often hold a disproportionate balance of power to achieve desirable economic, social, health or other outcomes. Marmot has suggested that such disempowerment processes may account for several strong social gradients in health and disease (Marmot, 2006; Marmot, 2015), including for mental health disorders(Williams, Costa, & Leavell, 2017); through this lens, ethnic disparities in psychosis risk could arise as a function of being exposed to greater social or cultural barriers in achieving autonomy and control over one's environment (Marmot, 2006; Marmot, 2015; Public Health England & UCL Institute of Health Equity, 2017).

In reality, we expect a complex interplay of such factors will contribute to such psychosocial disempowerment processes, and further observational and experimental studies are required to replicate our findings, investigate potential psychosocial mechanisms and understand if they are associated with neurobiological signatures relevant to psychosis (Howes & Kapur, 2014). There is already some evidence that outsider status [as indexed via migrant status (Egerton et al., 2017), childhood trauma (Egerton et al., 2016; Oswald et al., 2014) or hearing impairment (Gevonden et al., 2014)] is associated with increased dopamine sensitisation in healthy individuals (Selten, Booij, Buwalda, & Meyer-Lindenberg, 2017); a mechanism important in pathogenesis of psychotic disorders, and is particularly sensitive to environmental insults (Howes & Kapur, 2009). If proven, our results would have import for the aetiology of psychotic disorders.

Our results also have the potential to inform public mental health strategies to prevent psychosis, by identifying individual and societal factors amenable to intervention. This may include universal strategies aimed at reducing structural inequalities in health, and selected strategies to protect vulnerable populations from experiencing exposure to factors which lead to psychosocial disempowerment (Arango et al., 2018). Our results are amongst the first to provide traction on factors which may drive excess rates of psychotic disorders in minority ethnic groups and, if replicated, potentially provide vital clues about ameliorable risk factors for intervention.

## Supplementary material

For supplementary material accompanying this paper visit https://doi.org/10.1017/S003329172000029X. click here to view supplementary material

## Group members

The European Network of National Schizophrenia Networks Studying Gene-Environment Interactions (EU-GEI) WP2 Group members include Ulrich Reininghaus, PhD, Department of Psychiatry and Neuropsychology, School for Mental Health and Neuroscience, South Limburg Mental Health Research and Teaching Network, Maastricht University Medical Centre, Maastricht, the Netherlands, and Department of Health Service and Population Research, Institute of Psychiatry, King's College London, De Crespigny Park, Denmark Hill, London, England; Kathryn Hubbard, MSc, Department of Health Service and Population Research, Institute of Psychiatry, King's College London, De Crespigny Park, Denmark Hill, London, England; Stephanie Beards, PhD, Department of Health Service and Population Research, Institute of Psychiatry, King's College London, De Crespigny Park, Denmark Hill, London, England; Simona A. Stilo, MSc, Department of Health Service and Population Research, and Department of Psychosis Studies, Institute of Psychiatry, King's College London, De Crespigny Park, Denmark Hill, London, England; Giada Tripoli, MSc, Department of Psychosis Studies, Institute of Psychiatry, King's College London, De Crespigny Park, Denmark Hill, London, England, and Department of Experimental Biomedicine and Clinical Neuroscience, Section of Psychiatry, University of Palermo, Palermo, Italy; Mara Parellada, MD, PhD, Department of Child and Adolescent Psychiatry, Hospital General Universitario Gregorio Marañón, School of Medicine, Universidad Complutense, Investigación Sanitaria del Hospital Gregorio Marañón (Centro de Investigación Biomédica en Red de Salud Mental), Madrid, Spain; Pedro Cuadrado, MD, Villa de Vallecas Mental Health Department, Villa de Vallecas Mental Health Centre, Hospital Universitario Infanta Leonor/Hospital Virgen de la Torre, Madrid, Spain; José Juan Rodríguez Solano, MD, Puente de Vallecas Mental Health Department, Hospital Universitario Infanta Leonor/Hospital Virgen de la Torre, Centro de Salud Mental Puente de Vallecas, Madrid, Spain; Angel Carracedo, MD, PhD, Fundación Pública Galega de Medicina Xenómica, Hospital Clínico Universitario, Santiago de Compostela, Spain; Enrique García Bernardo, MD, Department of Psychiatry, Hospital General Universitario Gregorio Marañón, School of Medicine, Universidad Complutense, Investigación Sanitaria del Hospital Gregorio Marañón (Centro de Investigación Biomédica en Red de Salud Mental), Madrid, Spain; Laura Roldán, PhD, Department of Child and Adolescent Psychiatry, Hospital General Universitario Gregorio Marañón, School of Medicine, Universidad Complutense, Investigación Sanitaria del Hospital Gregorio Marañón (Centro de Investigación Biomédica en Red de Salud Mental), Madrid, Spain; Gonzalo López, PhD, Department of Child and Adolescent Psychiatry, Hospital General Universitario Gregorio Marañón, School of Medicine, Universidad Complutense, Investigación Sanitaria del Hospital Gregorio Marañón (Centro de Investigación Biomédica en Red de Salud Mental), Madrid, Spain; Bibiana Cabrera, MSc, PhD, Barcelona Clinic Schizophrenia Unit, Neuroscience Institute, Hospital Clinic of Barcelona. Barcelona. Spain. CIBERSAM, Spain; Esther Lorente-Rovira, PhD, Department of Psychiatry, School of Medicine, Universidad de Valencia, Centro de Investigación Biomédica en Red de Salud Mental, Valencia, Spain; Paz Garcia-Portilla, MD, PhD, Department of Medicine, Psychiatry Area, School of Medicine, Universidad de Oviedo, Centro de Investigación Biomédica en Red de Salud Mental, Oviedo, Spain; Javier Costas, PhD, Fundación Pública Galega de Medicina Xenómica, Hospital Clínico Universitario, Santiago de Compostela, Spain; Estela Jiménez-López, MSc, Department of Psychiatry, Servicio de Psiquiatría Hospital ‘Virgen de la Luz’, Cuenca, Spain; Mario Matteis, MD, Department of Child and Adolescent Psychiatry, Hospital General Universitario Gregorio Marañón, School of Medicine, Universidad Complutense, Investigación Sanitaria del Hospital Gregorio Marañón (Centro de Investigación Biomédica en Red de Salud Mental), Madrid, Spain; Marta Rapado, PhD, Department of Child and Adolescent Psychiatry, Hospital General Universitario Gregorio Marañón, School of Medicine, Universidad Complutense, Investigación Sanitaria del Hospital Gregorio Marañón (Centro de Investigación Biomédica en Red de Salud Mental), Madrid, Spain; Emiliano González, PhD, Department of Child and Adolescent Psychiatry, Hospital General Universitario Gregorio Marañón, School of Medicine, Universidad Complutense, Investigación Sanitaria del Hospital Gregorio Marañón (Centro de Investigación Biomédica en Red de Salud Mental), Madrid, Spain; Covadonga Martínez, MD, Department of Child and Adolescent Psychiatry, Hospital General Universitario Gregorio Marañón, School of Medicine, Universidad Complutense, Investigación Sanitaria del Hospital Gregorio Marañón (Centro de Investigación Biomédica en Red de Salud Mental), Madrid, Spain; Emilio Sánchez, MD, Department of Psychiatry, Hospital General Universitario Gregorio Marañón, School of Medicine, Universidad Complutense, Investigación Sanitaria del Hospital Gregorio Marañón (Centro de Investigación Biomédica en Red de Salud Mental), Madrid, Spain; Mª Soledad Olmeda, MD, Department of Psychiatry, Hospital General Universitario Gregorio Marañón, School of Medicine, Universidad Complutense, Investigación Sanitaria del Hospital Gregorio Marañón (Centro de Investigación Biomédica en Red de Salud Mental), Madrid, Spain; Nathalie Franke, MSc, Department of Psychiatry, Early Psychosis Section, Academic Medical Centre, University of Amsterdam, Amsterdam, the Netherlands; Jean-Paul Selten, MD, PhD, Department of Psychiatry and Neuropsychology, School for Mental Health and Neuroscience, Maastricht University Medical Centre, Maastricht, the Netherlands, and Rivierduinen Institute for Mental Health Care, Leiden, the Netherlands; Fabian Termorshuizen, PhD, Department of Psychiatry and Neuropsychology, School for Mental Health and Neuroscience, South Limburg Mental Health Research and Teaching Network, Maastricht University Medical Centre, Maastricht, the Netherlands, and Rivierduinen Centre for Mental Health, Leiden, the Netherlands; Daniella van Dam, PhD, Department of Psychiatry, Early Psychosis Section, Academic Medical Centre, University of Amsterdam, Amsterdam, the Netherlands; Elles Messchaart, MSc, Rivierduinen Centre for Mental Health, Leiden, the Netherlands; Marion Leboyer, MD, PhD, AP-HP, Groupe Hospitalier ‘Mondor’, Pôle de Psychiatrie, Créteil, France, Institut National de la Santé et de la Recherche Médicale, U955, Créteil, France, Faculté de Médecine, Université Paris-Est, Créteil, France, and Fondation Fondamental, Créteil, France; Franck Schürhoff, MD, PhD, AP-HP, Groupe Hospitalier ‘Mondor’, Pôle de Psychiatrie, Créteil, France, Institut National de la Santé et de la Recherche Médicale, U955, Créteil, France, Faculté de Médecine, Université Paris-Est, Créteil, France, and Fondation Fondamental, Créteil, France; Stéphane Jamain, PhD, Institut National de la Santé et de la Recherche Médicale, U955, Créteil, France, Faculté de Médecine, Université Paris-Est, Créteil, France, and Fondation Fondamental, Créteil, France; Flora Frijda, MSc, Etablissement Public de Santé Maison Blanche, Paris, France; Grégoire Baudin, MSc, AP-HP, Groupe Hospitalier ‘Mondor’, Pôle de Psychiatrie, Créteil, France, Institut National de la Santé et de la Recherche Médicale, U955, Créteil, France; Aziz Ferchiou, MD, AP-HP, Groupe Hospitalier ‘Mondor’, Pôle de Psychiatrie, Créteil, France, Institut National de la Santé et de la Recherche Médicale, U955, Créteil, France; Baptiste Pignon, MD, AP-HP, Groupe Hospitalier ‘Mondor’, Pôle de Psychiatrie, Créteil, France, Institut National de la Santé et de la Recherche Médicale, U955, Créteil, France, and Fondation Fondamental, Créteil, France; Jean-Romain Richard, MSc, Institut National de la Santé et de la Recherche Médicale, U955, Créteil, France, and Fondation Fondamental, Créteil, France; Thomas Charpeaud, MD, Fondation Fondamental, Créteil, France, CMP B CHU, Clermont Ferrand, France, and Université Clermont Auvergne, Clermont-Ferrand, France; Anne-Marie Tronche, MD, Fondation Fondamental, Créteil, France, CMP B CHU, Clermont Ferrand, France, and Université Clermont Auvergne, Clermont-Ferrand, France; Pierre-Michel Llorca MD, PhD, EA 7280 Npsydo, Université Clermont Auvergne, Clermont-Ferrand, France; Daniele La Barbera, MD, PhD, Department of Experimental Biomedicine and Clinical Neuroscience, Section of Psychiatry, University of Palermo, Palermo, Italy; Giovanna Marrazzo, MD, PhD, Unit of Psychiatry, ‘P. Giaccone’ General Hospital, Palermo, Italy; Lucia Sideli, PhD, Department of Experimental Biomedicine and Clinical Neuroscience, Section of Psychiatry, University of Palermo, Palermo, Italy; Crocettarachele Sartorio, PhD, Unit of Psychiatry, ‘P. Giaccone’ General Hospital, Palermo, Italy; Laura Ferraro, PhD, Department of Experimental Biomedicine and Clinical Neuroscience, Section of Psychiatry, University of Palermo, Palermo, Italy; Fabio Seminerio, MSc, Department of Experimental Biomedicine and Clinical Neuroscience, Section of Psychiatry, University of Palermo, Palermo, Italy; Cristina-Marta Del-Ben, MD, PhD, Division of Psychiatry, Department of Neuroscience and Behaviour, Ribeirão Preto Medical School, University of São Paulo, São Paulo, Brazil; Camila Marcelino Loureiro, MD, Departamento de Neurociências e Ciencias do Comportamento, Faculdade de Medicina de Ribeirão Preto, Universidade de São Paulo, São Paulo, Brasil, and Núcleo de Pesquina em Saúde Mental Populacional, Universidade de São Paulo, São Paulo, Brasil; Rosana Shuhama, PhD, Departamento de Neurociências e Ciencias do Comportamento, Faculdade de Medicina de Ribeirão Preto, Universidade de São Paulo, São Paulo, Brasil, and Núcleo de Pesquina em Saúde Mental Populacional, Universidade de São Paulo, São Paulo, Brasil; Federico Suprani, MD, Transcultural Psychosomatic Tream (BoTPT), Department of Surgical and Medical Sciences, Bologna University, Bologna, Italy; Mirella Ruggeri, MD, PhD, Section of Psychiatry, Department of Neuroscience, Biomedicine and Movement, University of Verona, Verona, Italy; Sarah Tosato, MD, PhD, Section of Psychiatry, Department of Neuroscience, Biomedicine and Movement, University of Verona, Verona, Italy; Chiara Bonetto, PhD, Section of Psychiatry, Department of Neuroscience, Biomedicine and Movement, University of Verona, Verona, Italy; and Doriana Cristofalo, MA, Section of Psychiatry, Department of Neuroscience, Biomedicine and Movement, University of Verona, Verona, Italy.
