# Supplementary material for: Social disadvantage, linguistic distance, ethnic minority status and first-episode psychosis: results from the EU-GEI case–control study
Source: Psychol Med. 2020 Mar 3;51(9):1536–48. doi: 10.1017/S003329172000029X (PMC8311819; doi:10.1017/S003329172000029X)
Supplement: Supplementary file 1 [file S003329172000029Xsup001.docx]

Supplemental materials to:

Jongsma HE, Gayer-Anderson C, Tarricone I, Velthorst E, van der Ven E, Quattrone D, … Kirkbride JB (2020) Social disadvantage, linguistic distance, ethnic minority status and first episode psychosis: results from the EU-GEI case-control study. *Psychological Medicine*

**Supplemental Tables**

[Supplemental Table 1: Ethnicity by country 3](#_Toc29389455)

[Supplemental Table 2: STROBE checklist 7](#_Toc29389456)

[Supplemental Table 3: Original analysis plan 8](#_Toc29389457)

[Supplemental Table 4: Representativeness of the control sample compared with the population-at-risk by catchment area 13](#_Toc29389458)

[Supplemental Table 5: Correlations between exposures and confounders in the control sample (n=1,495) 14](#_Toc29389459)

[Supplemental Table 6: Odds of psychotic disorders by generational status following incremental covariate adjustment 16](#_Toc29389460)

[Supplemental Table 7: Odds of non-affective psychotic disorder by exposure status following incremental covariate adjustment (n=2,256) 17](#_Toc29389461)

[Supplemental Table 8: Odds of affective psychotic disorders by exposure status following incremental covariate adjustment (n=1,801) 18](#_Toc29389462)

[Supplemental Table 9: Sensitivity analyses of the associations between FEP risk and ethnic group, by statistical model (complete cases only, using inverse probability weights) 19](#_Toc29389463)

**Supplemental Figures**[Supplemental Figure 1: Language families and branches included in the EU-GEI study **.* 4](#_Toc29303667)

[Supplemental Figure 2: Distribution of language distance 5](#_Toc29303668)

[Supplemental Figure 3: Distribution of self-rated fluency 5](#_Toc29303669)

[Supplemental Figure 4: Distribution of linguistic distance by ethnic group 15](#_Toc29303670)

[Supplemental Figure 5: Distribution of linguistic distance by generational status 15](#_Toc29303671)

**Supplemental Methods**

*Purposive oversampling*

We attempted to purposively over-sample some traditionally hard to reach control groups, such as young BME men, in some settings using *ad hoc* targeted recruitment strategies including internet and newspaper adverts, and leaflets at local stations, shops and job centres. Nonetheless, our controls were broadly representative of the population at-risk by sex and ethnicity (Supplemental Table 1), and we used inverse probability weighting in our sensitivity analyses (see below).

*Definition of ethnicity*

Ethnicity was defined differently in each country. Supplemental Table 1 details the main ethnic minority groups in England, the Netherlands, France and Brazil for each of the categories used in this paper. Italy and Spain are not included, as country of birth was used here. Italy and Spain have a relatively recent migration history, with few second-generation migrants over the age of 18. In England, we used a combination of self-ascribed ethnicity (categories as per the 2011 Census) and place of birth. In Supplemental Table 1, the census categories are mapped onto the categories used in this paper. Place of birth was only used to parse the Black African group in North-African (e.g. born in Tunisia) and Black (e.g. born in Ghana) and the Arab group in North-African (e.g. born in Egypt) and other (e.g. born in Saudi Arabia). In the Netherlands, someone is considered native-Dutch (White Dutch here) if they are born in the Netherlands to two Dutch-born parents. Country of birth of the participants and their parents was used to delineate common migrant groups (see Supplemental Table 1) and to divide the commonly used categories of ‘Western’ and ‘non-Western’ immigrant into the categories used in this paper. In France we used a combination of self-ascribed ethnicity and country of birth (see Supplemental Table 1). In Brazil, we ascribed ethnicity by skin colour, in line with Brazilian custom.

**Supplemental Table 1: Ethnicity by country**

| *Ethnic group* | *Country* | | | | | |
| --- | --- | --- | --- | --- | --- | --- |
|  | **England** | **Netherlands** | | **France** | **Brazil** | |
| White majority | White British | White Dutch | | White French | White | |
| Black | Black Caribbean, Black African, Other Black | Surinamese, Antillian | | Black African, Antillian | Black | |
| Mixed | Any mixed ethnic background | Any mixed ethnic background | | Any mixed Background | Brown, Indigenous | |
| Asian | Indian, Pakistani, Bangladeshi, Chinese, Other Asian | Non-Western immigrants | | Asian | Yellow | |
| North-African | Arab, Black African | Turkish, Moroccan | | Arab (Maghreb) | n/a | |
| White minority | White Irish, Other White | Western immigrants | | Other European, American | White | |
| Other | Other, Arab | Non-Western immigrants | Jewish, other | | | n/a |

*Composition of language distance*

Language distance was composed using a simplified language tree (Supplemental Figure 1), adapted to the languages spoken by our participants, where language families were subdivided into branches. Scores were created as follows:

- 0: first language is the same as the majority language. An example is an English speaker living in England
- 1: first language was on the same branch in the same family. An example is a Dutch speaker living in England (both Germanic languages in the Indo-European family).
- 2: first language was on a different branch in the same family. An example is a French speaker living in England (French is on the Italic branch of the Indo-European family, English is on the Germanic branch).
- 3: first language is in a different language family altogether. An example would be a Korean speaker living in England.

If participants indicated that a local language or dialect was their first language, they were classed as a 0. In practice, this only occurred in some Spanish settings (i.e. Catalonian in Barcelona).

Due to the skewness in both language distance (Supplemental Figure 2) and fluency (Supplemental Figure 3), a binary measure of linguistic distance was used.

**Supplemental Figure 1: Language families and branches included in the EU-GEI study** **.*

English and Dutch are Germanic languages (in blue).

French, Italian, Spanish and Portuguese are Italic languages (in red).


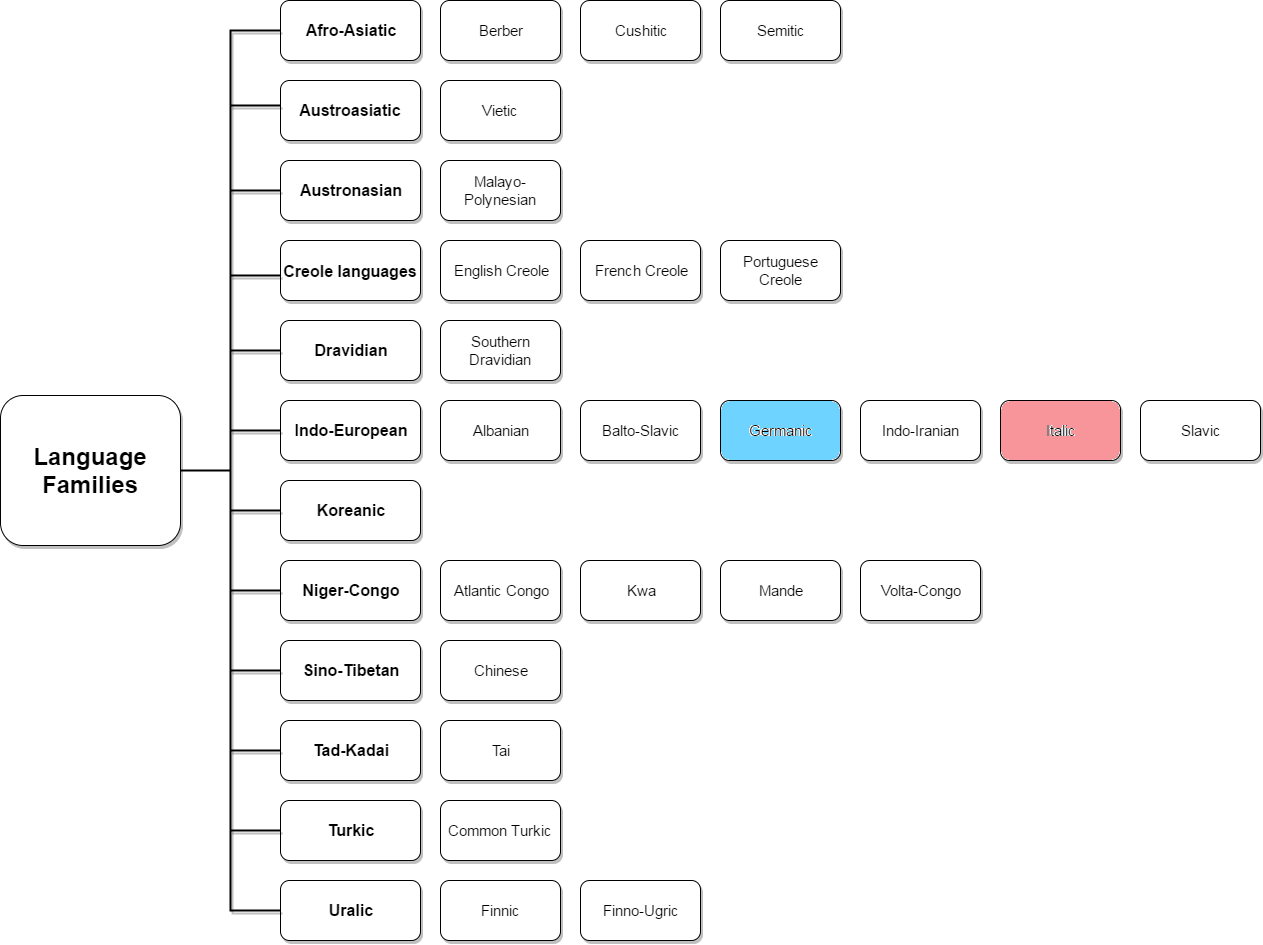


* Please note this is not a complete language tree. It only includes languages spoken by EU-GEI participants. Language families and branches are ordered alphabetically.

**Supplemental Figure 2: Distribution of language distance**

A language distance of **0** indicates a participant’s first language is the majority language of their country of residence.
A language distance of **1** indicates a participant’s first language is on the same branch of the same language family as the majority language in their country of residence (i.e. a Dutch speaker living in England).
A language distance of **2** indicates a participant’s first language is on a different branch of the same language family as the majority language in their country of residence (i.e. a Spanish speaker living in England).
A language distance of **3** indicates a participant’s first language is in a different language family as the majority language in their country of residence (i.e. a Korean speaker living in England)

**Supplemental Figure 3: Distribution of self-rated fluency**

*Discrimination*

The discrimination questionnaire consisted of the following items:

For any reason, have you ever been unfairly…

1. Fired
2. Not hired for a job
3. Denied promotion
4. Stopped, questioned, threatened by police
5. Treated by the court system
6. Discouraged from continuing education
7. Prevented from buying, renting flat or house
8. Treated by neighbours or your family
9. Denied a loan or preferable mortgage rate
10. Received worse service than other people
11. Treated when getting medical care
12. Treated when using public transport

If participants responded ‘yes’ on any item, they were asked the number of times this had happened, the age at which this had first occurred and the perceived main reason for this. Answering options for the latter were gender, race/ethnicity, religion, mental illness, sexuality, age, other (please specify). For the purposes of this study, we used the number of items participants endorsed (the number of ‘yes’ answers), regardless of the frequency of this happening. This corresponds to a score ranging from 0 to 12.

*Missing data*

In order to avoid dropping observations with missing data, we used multiple imputation. To increase the likelihood of satisfying the assumption that data was Missing At Random (MAR), the imputation model included all variables listed in the main analyses, as well as several auxiliary variables(Sullivan et al., 2016). The following variables had missing values and were imputed: age, self-perceived discrimination, paternal socioeconomic status, level of education, relationship status, living arrangements, cannabis use, childhood trauma, parental history of mental illness and paternal age. Case-control status, sex, ethnicity, linguistic distance, diagnosis (non-affective, affective, psychosis NOS), Wechsler Adult Intelligence Scale score, neighbourhood trust (measured by Social Environment Assessment Tool), first language, years in education, religion and frequency of religious service attendance were used as auxiliary variables.

*Weighting and sensitivity analyses*

To account for any over- or under-sampling of controls relative to the population at-risk (see Supplemental Table 1), we used inverse probability weights; we gave each control’s data a weight inversely proportional to their probability of selection on key demographics (age, gender, binary majority/minority ethnicity status) using census data on relevant populations. These weights were applied in sensitivity analyses, based on the complete-case sample.

*STROBE checklist*

The full STROBE checklist can be found in Supplemental Table 1 below. The only item not explicitly addressed elsewhere in the paper or the supplemental material is the sample size calculation. The EU-GEI study was powered to ‘recruit a sample of 1,200 incident cases, 600 siblings and 1,200 controls. Given the population covered, these are conservative estimates (i.e., equates to approximately 16-18 anticipated cases per 100,000 at risk per year – well within previous reported rates).’(European Network of National Schizophrenia Networks Studying Gene-Environment Interactions, 2013)

**Supplemental Table 2: STROBE checklist**

|  | | **Item No** | **Recommendation** | **Achieved** |
| --- | --- | --- | --- | --- |
| **Title and abstract** | | 1 | (*a*) Indicate the study’s design with a commonly used term in the title or the abstract | p.1,5,6 |
|  |  |  | (*b*) Provide in the abstract an informative and balanced summary of what was done and what was found | p.5,6 |
| **Introduction** | | | |  |
| Background / rationale | | 2 | Explain the scientific background and rationale for the investigation being reported | p.7 |
| Objectives | | 3 | State specific objectives, including any prespecified hypotheses | p.8 |
| **Methods** | | | |  |
| Study design | | 4 | Present key elements of study design early in the paper | p.8,9 |
| Setting | | 5 | Describe the setting, locations, and relevant dates, including periods of recruitment, exposure, follow-up, and data collection | p.8 |
| Participants | | 6 | *Case-control study*—Give the eligibility criteria, and the sources and methods of case ascertainment and control selection. Give the rationale for the choice of cases and controls | p.8,9 |
|  |  |  | *Case-control study*—For matched studies, give matching criteria and the number of controls per case | n/a |
| Variables | | 7 | Clearly define all outcomes, exposures, predictors, potential confounders, and effect modifiers. Give diagnostic criteria, if applicable | p.9,10 |
| Data sources / measurement | | 8 | For each variable of interest, give sources of data and details of methods of assessment (measurement). Describe comparability of assessment methods if there is more than one group | p.9,10 |
| Bias | | 9 | Describe any efforts to address potential sources of bias | p.10,11 |
| Study size | | 10 | Explain how the study size was arrived at | See above |
| Quantitative variables | | 11 | Explain how quantitative variables were handled in the analyses. If applicable, describe which groupings were chosen and why | p. 10,11 |
| Statistical methods | | 12 | (*a*) Describe all statistical methods, including those used to control for confounding | p.10,11 |
|  |  |  | (*b*) Describe any methods used to examine subgroups and interactions | p.11 |
|  |  |  | (*c*) Explain how missing data were addressed | p.10 |
|  |  |  | *Case-control study*—If applicable, explain how matching of cases and controls was addressed | n/a |
|  |  |  | (*e*) Describe any sensitivity analyses | p. 11 |
| Results | | | |  |
| Participants | 13 | (a) Report numbers of individuals at each stage of study—eg numbers potentially eligible, examined for eligibility, confirmed eligible, included in the study, completing follow-up, and analysed | | Reported in detail elsewhere, referenced in text on p.11,12 |
|  |  | (b) Give reasons for non-participation at each stage | |  |
|  |  | (c) Consider use of a flow diagram | |  |
| Descriptive data | 14 | (a) Give characteristics of study participants (eg demographic, clinical, social) and information on exposures and potential confounders | | Table 1 |
|  |  | (b) Indicate number of participants with missing data for each variable of interest | | Table 1 |
| Outcome data | 15 | *Case-control study—*Report numbers in each exposure category, or summary measures of exposure | | Table 3 |
| Main results | 16 | (*a*) Give unadjusted estimates and, if applicable, confounder-adjusted estimates and their precision (eg, 95% confidence interval). Make clear which confounders were adjusted for and why they were included | | Table 3 |
|  |  | (*b*) Report category boundaries when continuous variables were categorized | | p. 9,10 |
|  |  | (*c*) If relevant, consider translating estimates of relative risk into absolute risk for a meaningful time period | | n/a |
| Other analyses | 17 | Report other analyses done—eg analyses of subgroups and interactions, and sensitivity analyses | | p.14 Table S2 |
| Discussion | | | |  |
| Key results | 18 | Summarise key results with reference to study objectives | | p.14 |
| Limitations | 19 | Discuss limitations of the study, taking into account sources of potential bias or imprecision. Discuss both direction and magnitude of any potential bias | | p. 14,15 |
| Interpretation | 20 | Give a cautious overall interpretation of results considering objectives, limitations, multiplicity of analyses, results from similar studies, and other relevant evidence | | p.16,17 |
| Generalisability | 21 | Discuss the generalisability (external validity) of the study results | | p.16,17 |
| Other information | | | |  |
| Funding | 22 | Give the source of funding and the role of the funders for the present study and, if applicable, for the original study on which the present article is based | | p.18 |
| Note: An Explanation and Elaboration article discusses each checklist item and gives methodological background and published examples of transparent reporting. The STROBE checklist is best used in conjunction with this article (freely available on the Web sites of PLoS Medicine at http://www.plosmedicine.org/, Annals of Internal Medicine at http://www.annals.org/, and Epidemiology at http://www.epidem.com/). Information on the STROBE Initiative is available at www.strobe-statement.org. | | | | |

**Supplemental Table 3: Original analysis plan**

This was the original analysis plan as submitted to the EU-GEI steering committee in summer 2016. Synopses are an internal mechanism to track studies across the project. The methods section is of most interest for the present purpose.

| **Synopsis no.:** S2.29 **NB this synopsis was approved by the EU-GEI team circa summer 2016.** |
| --- |
| **Preliminary title: Social and cultural distance as an explanation of higher rates of psychotic disorders in minority groups** |
| **Contact info for the person(s) proposing the synopsis**  **Name:** Hannah Jongsma **Partner no:** 23  **e-mail address:** hej33@cam.ac.uk |
| **Publication category: Peer reviewed publication** |
| **Working and writing group:** Peter Jones, James Kirkbride, Charlotte Gayer-Anderson, Craig Morgan |
| **Work Packages involved:** WP2 |
| **Partners involved from whom candidate co-authors (additional to working and writing group) should be nominated:** Jean-Paul Selten |
| **Objectives (scientific background, hypothesis, methods, and expected results):**    **Background**  Rates of psychotic disorders are consistently higher in ethnic minority groups^1^ but different ethnic groups appear to be affected in different host countries, such as those of Moroccan ancestry in the Netherlands^2^, and Black African and Black Caribbean groups in the UK^3^. This risk appears to be specific to individuals who have migrated to Western countries as rates in the general population in for instance Jamaica^4^ and Surinam^5^ appear to be similar to rates in the general population in Western Europe. Furthermore, within the same ethnic groups, first, second and even third generations can have differential risks^6^.    This is perhaps unsurprising when considering the sociological argument that ethnicity is not a discreet variable: it doesn’t have the same meaning to everyone^7^. This points to a more complex explanation for the higher rates of disorder in ethnic minority groups^8^. As psychotic disorders are a disorder of social functioning, including the loss of social-cognitive skills and alienation of the self from the social world^9^, we propose to look at the position minorities hold in society. Initially, this exploration will focus on ethnic minority groups, but we will also tentatively examine religious minority groups. We suggest that minority groups experience higher levels of what Michael Marmot names psychosocial disempowerment: having control over your life^10^. Minority populations tend to be lower down the social ladder, and social hierarchy in itself is a stressor^11^, but we suggest the effects of psychosocial disempowerment are additive to this. This theory differs from the social defeat theory^8^ as the focus is on social and cultural distance from the majority, as a continual underlying stressor, and not the more direct notion of defeat.    We arrive at this theory employing a more economic argument, in the following steps:   1. Choosing and being able to express one’s identity is of fundamental importance to maximising utility or well-being ^12^. 2. Minorities experience more restrictions on being able to do this, for two reasons: 3. Boundaries of the identity are more externally determined: set by the majority population ^[[1]](#footnote-1)213^, and 4. Forming and expressing an identity is a very social process and is largely facilitated by complex forms of imitation^14^; it is less clear which group minority groups should look to imitate for forming a distinct identity. 5. Empathy in society comes from a shared sense of identity. This empathy leads to mutual regard or trust, and this extends to fellow citizens^15^. 6. It is proposed that the difficulties minorities experience in forming and expressing their identity places them to an extent outside the group op ‘fellow citizens’, and that it is this distance from the majority population that increases psychosocial disempowerment, which in turn increases the risk of psychotic disorders.     We will investigate this using the following key concepts (how this is measured within EU-GEI can be found under the ‘methods’ and ‘data needed’ sections):   - Psychosocial disempowerment: the feeling of not having control over one’s life - Social distance: Essentially an elaboration of social disadvantage, also looking at educational attainment and social isolation. This will increase daily life stresses for minority groups, and places them outside society. The distance is from those who hold the most economic and political resources (least disadvantaged). The concept is different from psychosocial disempowerment in that it looks at material and social factors, not at the subjective experience of the consequences of these. - Cultural distance: The extent to which ethnic minority groups come from cultural backgrounds that are different from the majority’s background. This only applies to ethnic minorities, and not to religious minorities.     The argument means that higher rates of psychotic disorders are not necessarily limited to ethnic minorities, but in Western societies these currently occupy a position of high psychosocial disempowerment. As identities are fluid and subject to change over time, it is possible that other groups will occupy this position in the future. We will also examine this argument more broadly, looking at religious minorities.    **Aims**  The overall aim of this synopsis is to disentangle the effects of psychosocial disempowerment and cultural and social distance on the higher rates of psychotic disorders in minority groups. In order to investigate this, we will test the following concrete hypotheses:     1. Cases are more likely to be of ethnic minority status than controls 2. Cases are more likely to be from more culturally distant ethnic minority groups than controls. 3. Increased cultural distance is positively associated with increased risk of developing psychotic disorders. 4. Cases report higher levels of psychosocial disempowerment than controls. 5. Those of ethnic minority status report higher levels of psychosocial disempowerment. 6. The larger the cultural and social distance, the higher the levels of psychosocial disempowerment. 7. Cumulative social and cultural distance has a similar relationship to higher rates of disorder in minority groups as cultural distance alone, but explains a higher proportion of the variance. 8. The relationship between ethnic minority status and higher risk of developing a psychotic disorder is mediated by cultural and social distance.     I also aim to explore the risk of developing a psychotic disorder in religious minority groups, and to test if this is also mediated by psychosocial disempowerment.    **Methods**  The following variables will be included (for a list of precise data items, see below)    Outcome variable: case/control/sibling status    Predictor variables: ethnic minority status, cultural distance, and psychosocial disempowerment.   - Ethnic minority status - Cultural distance is closely correlated with linguistic distance^15^, and I will use language trees to approximate this. - Social distance will consist of socio-economic status, years of education, cognitive distance and social isolation. Cognitive distance will be approximated using performance on the Wechsler Adult Intelligence Scale (WAIS) by taking account of standard deviations away from the country’s mean. - Psychosocial disempowerment will be measured using self-perceived discrimination, and generalised neighbourhood trust. When looking at ethnic minorities, perceptions of ethnic diversity and cooperation in the neighbourhood are also taken into account. - An individual is considered a religious minority if they’re any religion other than Christian.     Confounding will be appropriately allowed for by including age, sex, family history of psychosis and other mental illness, cannabis use, childhood trauma, and paternal age.    **Expected results**  The expected results can be found in the aims of the study. Overall, we expect that social and cultural distance and psychosocial disempowerment mediate the relationship between (ethnic) minority status and risk of psychotic disorders.    When looking at religious minorities, it is expected that the role of cultural distance diminishes, but the role of social distance and psychosocial disempowerment remains similar.  **Plan for statistical analysis (overall strategy):**  Data will be cleaned in cooperation with Charlotte Gayer-Anderson and Marta di Forti (IoPPN).    Univariate associations will be tested using univariate logistic regression. This will subsequently be expanded into a multivariate model, and into a regression model allowing for the mediating effect of cultural and social distance    Methods will be further developed in cooperation with James Kirkbride and Peter Jones.  **Timeframe:**  We aim to have all the data cleaned and variables created by 1 September, and to have the analyses completed by 1 October. An initial draft of results will be available by 1 January 2017.  **Additional comments:**  This synopsis is part of a PhD-project looking at an explanatory framework for higher rates of psychotic disorders in minorities using EU-GEI data, under supervision of James Kirkbride and Peter Jones.    **Bibliography**   1. Bourque F, van der Ven E, Malla A. A meta-analysis of the risk for psychotic disorders among first- and second-generation immigrants. Psychol Med. 2011;41(5):897-910.   doi:10.1017/S0033291710001406.   1. Veling W, Selten JP, Veen N, Laan W, Blom JD, Hoek HW. Incidence of schizophrenia among ethnic minorities in the Netherlands: A four-year first-contact study. Schizophr Res. 2006;86(13):189-193. doi:10.1016/j.schres.2006.06.010. 2. Morgan C, Dazzan P, Morgan K, et al. First episode psychosis and ethnicity: initial findings from the AESOP study. World Psychiatry. 2006;5(1):40-46.   http://www.pubmedcentral.nih.gov/articlerender.fcgi?artid=1472260&tool=pmcentrez&renderty pe=abstract.   1. Hickling FW. The incidence of first-contact schizophrenia in Jamaica. Br J Psychiatry. 1995;167(December 1992):193-196. 2. Hanoeman M, Selten J-P, Kahn RS. Incidence of schizophrenia in Surinam. Schizophr Res. 2002;54(3):219-221. doi:10.1016/S0920-9964(01)00269-9. 3. Oh H, Abe J, Negi N, Devylder J. Immigration and psychotic experiences in the United States: Another example of the epidemiological paradox? Psychiatry Res. 2015;229:784-790.   doi:10.1016/j.psychres.2015.08.002.   1. Fenton S, Charlsley K. Epidemiology and sociology as incommensurate games: accounts from the study of health and ethnicity. Health (Irvine Calif). 2000;4(4):403-425. 2. Selten JP, Van Der Ven E, Rutten BPF, Cantor-Graae E. The social defeat hypothesis of schizophrenia: An update. Schizophr Bull. 2013;39(6):1180-1186. doi:10.1093/schbul/sbt134. 3. Abed RT, Abbas MJ. A reformulation of the social brain theory for schizophrenia: the case for out-group intolerance. Perspect Biol Med. 2011;54(2):132-151. doi:10.1353/pbm.2011.0020. 4. Marmot M. The Health Gap. London: Bloomsbury Publishing; 2015. 5. Sapolsky RM. The influence of social hierarchy on primate health. Science. 2005;308(5722):648652. doi:10.1126/science.1106477. 6. Akerlof G, Kranton R. Identity Economics: How Our Identities Shape Our Work, Wages and Well-Being. Princeton, New Jersey: Princeton University Press; 2011. 7. N H. Ethnic Minority Identity: A Social Psychological Perspective. Oxford: Clarendon Press; 1992. 8. Dijksterhuis A. Why We Are Social Animals: The High Road to Imitation as Social glue. In: Hurley S, Chater N, eds. Imitation, Human Development, and Culture. Cambridge, Massachusetts: The MIT Press; 2005. 9. Collier P. Exodus: How Migration Is Changing Our World. New York: Oxford University Press; 2013. |

**Supplemental Results**

*Representativeness of the control sample*

We tested the degree to which the controls were representative of the population at-risk using population Census data in each catchment area, as previously described (Jongsma et al., 2018). Overall (Supplemental Table 3) controls were representative of the population at-risk in terms of ethnic minority status and sex. Nonetheless, in Val-de-Marne ethnic minority groups were over-sampled, and in Gouda & Voorhout and Bologna they were under-sampled. In Bologna there was some evidence women were over-sampled. Younger people (under 35 years old) were over-represented overall compared with the population at-risk, largely driven by recruitment of a higher proportion of younger controls in catchment areas in Southeast London, Amsterdam, Italy, France and Brazil (Supplemental Table 3).

**Supplemental Table 4: Representativeness of the control sample compared with the population-at-risk by catchment area**

|  | Age | | | | Sex | | | | Ethnic majority status | | | |  |
| --- | --- | --- | --- | --- | --- | --- | --- | --- | --- | --- | --- | --- | --- |
|  | 18-34 year olds | |  |  | Men | |  |  | Majority status | |  |  |  |
|  | Population | Controls |  |  | Population | Controls |  |  | Population | Controls |  |  |  |
|  | *n* (%) | *n*  (%) | χ^2^ | p-value | *n* (%) | *n* (%) | χ^2^ | p-value | *n* (%) | *n* (%) | χ^2^ | p-value |  |
| England |  |  |  |  |  |  |  |  |  |  |  |  |  |
| Southeast London | 214,713 (50.4) | 155 (67.4) | **26.71** | **<0.0001** | 212,918 (49.9) | 113 (49.1) | 0.06 | 0.806 | 175,706 (41.2) | 105 (45.7) | 1.86 | 0.173 |  |
| Cambridgeshire | 582,569 (37.5) | 37 (34.9) | 0.32 | 0.583 | 782,607 (50.4) | 50 (47.2) | 0.42 | 0.513 | 1,238,172 (79.7) | 85 (80.2) | 0.02 | 0.891 |  |
| The Netherlands |  |  |  |  |  |  |  |  |  |  |  |  |  |
| Amsterdam | 256,659 (41.3) | 53 (52.5) | **5.20** | **0.023** | 313,287 (50.4) | 47 (46.5) | 0.61 | 0.433 | 293,709 (47.3) | 57 (56.4) | 3.42 | 0.061 |  |
| Gouda & Voorhout | 242,922 (31.2) | 38 (34.9) | 0.51 | 0.475 | 384,975 (50.2) | 52 (47.7) | 0.27 | 0.602 | 651,786 (85.0) | 102 (93.6) | **6.28** | **0.012** |  |
| Spain |  |  |  |  |  |  |  |  |  |  |  |  |  |
| Madrid | 149.670 (36.1) | 16 (43.2) | 0.82 | 0.364 | 205,364 (49.5) | 18 (48.7) | 0.01 | 0.916 | 329,424 (79.4) | 28 (75.7) | 0.32 | 0.573 |  |
| Barcelona | 311,664 (35.3) | 14 (37.5) | 0.11 | 0.743 | 426,259 (48.2) | 16 (43.2) | 0.37 | 0.544 | 299,983 (82.37) | 27 (72.97) | 0.51 | 0.473 |  |
| Valencia | 125,239 (34.4) | 10 (32.3) | 0.06 | 0.803 | 180,696 (49.6) | 14 (45.2) | 0.25 | 0.620 | 428,482 (92.6) | 25 (80.7) | 0.06 | 0.801 |  |
| Oviedo | 253,358 (30.8) | 23 (56.0) | **14.55** | **<0.0001** | 226,980 (49.1) | 19 (48.7) | <0.01 | 0.966 | 556,193 (96.7) | 33 (84.6) | 3.66 | 0.056 |  |
| Santiago | 189,135 (32.9) | 11 (29.0) | 0.27 | 0.604 | 286,767 (49.9) | 18 (47.4) | 0.10 | 0.757 | 160,723 (82.4) | 37 (97.4) | 0.05 | 0.826 |  |
| Cuenca | 68,672 (35.2) | 15 (39.5) | 0.30 | 0.582 | 102,695 (52.6) | 20 (52.6) | <0.01 | 1.00 | 179,220 (66.8) | 31 (81.6) | 0.02 | 0.895 |  |
| France |  |  |  |  |  |  |  |  |  |  |  |  |  |
| Val-de-Marne | 198,767 (38.9) | 50 (50.0) | **5.16** | **0.023** | 242,333 (47.5) | 47 (47.0) | <0.01 | 0.926 | 342,090 (67.0) | 51 (51.0) | **11.56** | **0.001** |  |
| Puy-de-Dome | 61,684 (27.2) | 22 (46.8) | **9.09** | **0.003** | 113,579 (50.1) | 21 (44.7) | 0.56 | 0.455 | 213,780 (94.4) | 43 (91.5) | 0.73 | 0.393 |  |
| Italy |  |  |  |  |  |  |  |  |  |  |  |  |  |
| Bologna | 272,987 (29.3) | 43 (66.2) | **47.62** | **<0.0001** | 455,829 (48.9) | 24 (36.9) | 3.74 | 0.053 | 727,700 (78.1) | 60 (92.3) | **7.67** | **0.006** |  |
| Veneto | 202,672 (40.1) | 65 (56.5) | **12.92** | **<0.0001** | 259,282 (51.3) | 53 (46.1) | 1.25 | 0.264 | 446,523 (88.3) | 102 (88.7) | 0.01 | 0.903 |  |
| Palermo | 541,472 (34.0) | 64 (64.0) | **40.26** | **<0.0001** | 781,004 (49.0) | 49 (49.0) | <0.01 | 1.00 | 1,493,856 (93.7) | 92 (92.0) | 0.47 | 0.494 |  |
| Brazil |  |  |  |  |  |  |  |  |  |  |  |  |  |
| Ribeirão Preto | 1,219,115 (46.3) | 216 (71.5) | **77.11** | **<0.0001** | 1,299,112 (49.4) | 144 (47.7) | 0.34 | 0.59 | 1,745,638 (66.3) | 204 (67.6) | 0.20 | 0.654 |  |
| Estimates in bold are significant (p<0.05). | | | | | | | | | | | | | |

*Correlations between exposures and confounders in the control sample*

Supplemental Table 5 details the polychoric correlations between the various exposures and confounds in the control sample (as a representation of the general population). The only strong correlations (coefficient of at least ± 0.6) are positive correlations between age and relationship status and between living arrangements (ever having lived with someone other than one’s parents) and relationship status. The only moderate correlations (coefficient of at least ± 0.4) a negative correlation between age and cannabis use and a positive correlation between age and living arrangements. Weak correlations (coefficient of at least ± 0.2) were observed between age and sex, sex and cannabis use, parental socioeconomic status and level of education and living arrangements and cultural distance (all positive), as well as sex and relationship status and cannabis use and parental socioeconomic status (both negative).

**Supplemental Table 5: Correlations between exposures and confounders in the control sample (n=1,495)**

| Variable | 1. | 2. | 3. | 4. | 5. | 6. | | 7. | 8. | 9. | 10. | 11. | 12 |
| --- | --- | --- | --- | --- | --- | --- | --- | --- | --- | --- | --- | --- | --- |
| 1. | 1 |  |  |  |  |  | |  |  |  |  |  |  |
| 2. | 0.25 | 1 |  |  |  |  | |  |  |  |  |  |  |
| 3. | 0.04 | 0.06 | 1 |  |  |  | |  |  |  |  |  |  |
| 4. | -0.45 | 0.20 | 0.00 | 1 |  |  | |  |  |  |  |  |  |
| 5. | 0.05 | -0.15 | -0.06 | 0.09 | 1 |  | |  |  |  |  |  |  |
| 6. | 0.06 | 0.06 | -0.16 | -0.05 | 0.04 | 1 | |  |  |  |  |  |  |
| 7. | 0.04 | 0.01 | -0.10 | -0.24 | 0.10 | 0.07 | | 1 |  |  |  |  |  |
| 8. | 0.00 | 0.00 | -0.01 | -0.18 | 0.15 | -0.06 | | 0.34 | 1 |  |  |  |  |
| 9. | 0.55 | -0.17 | -0.07 | -0.03 | 0.16 | -0.06 | | 0.01 | -0.16 | 1 |  |  |  |
| 10. | 0.63 | -0.22 | -0.14 | 0.07 | 0.12 | -0.08 | | 0.11 | -0.10 | 0.63 | 1 |  |  |
| 11. | -0.02 | -0.13 | 0.02 | -0.02 | 0.08 | -0.11 | | -0.18 | -0.19 | 0.23 | 0.08 | 1 |  |
| 12. | 0.10 | 0.06 | 0.05 | 0.15 | 0.19 | 0.01 | | -0.02 | -0.10 | 0.06 | 0.14 | 0.18 | 1 |
| 1. Age  2. Sex  3. Paternal age  4. Cannabis use  5. Childhood trauma  6. Parental history of psychosis | | | | | | | 7. Parental socioeconomic status  8. Level of education  9. Living arrangements  10. Relationship status  11. Cultural distance  12. Discrimination | | | | | | |

*Linguistic distance by ethnic group and generational status*

The distribution of linguistic distance by ethnic group and generational can be found in Supplemental Figures 4 and 5 respectively. Linguistic distance was most present in the Asian and North-African groups, and least in the White majority and mixed ethnic groups. First generation ethnic minorities had a higher linguistic distance compared with second-generation ethnic minorities.

**Supplemental Figure 4: Distribution of linguistic distance by ethnic group**

**Supplemental Figure 5: Distribution of linguistic distance by generational status**

*Risk in first- versus later-generation migrant groups*

Supplemental Table 6 details the odds ratios of psychotic disorders in first- and later-generation minorities compared with the white majority population. Crude excess odds were similar for generations (OR 1.91, 95%CI 1.52-2.39 for first generations and OR:2.15, 95%CI: 1.69-2.73 for second generations) and are attenuated but remain increased after adjustment for *a priori* confounders. Incremental adjustment for other covariates suggested that later-generation groups were no longer at increased odds of psychosis after adjustment for social disadvantage (Model B: OR: 1.24; 95%CI: 0.93-1.66), but risk remained elevated for first-generation migrants (OR: 1.82; 95%CI: 1.39-2.38) until further adjustment for linguistic distance (Model C: OR:1.28; 95%CI: 0.89-1.83; Supplemental Table 4/Figure 1).

**Supplemental Table 6: Odds of psychotic disorders by generational status following incremental covariate adjustment**

|  | Crude | Model A^1^ | Model B^2^ | Model C^3^ | Model D^4^ |
| --- | --- | --- | --- | --- | --- |
| Variable | OR (95%CI) | OR (95%CI) | OR (95%CI) | OR (95%CI) | OR (95%CI) |
| Generational status  First generation  Later generations | **1.91 (1.52-2.39)**  **2.15 (1.69-2.73)** | **1.65 (1.29-2.13)**  **1.53 (1.17-2.01)** | **1.82 (1.39-2.38)**  1.24 (0.92-1.66) | 1.28 (0.89-1.83)  1.20 (0.89-1.61) | 1.26 (0.88-1.82)  1.18 (0.88-1.59) |
| ^1^ Model A is adjusted for covariates (age, sex, their interaction, paternal age, childhood trauma, cannabis use, parental history of psychosis)  ^2^ Model B is further adjusted for indicators of social disadvantage (paternal SES, level of education, relationship status and living arrangements)  ^3^ Model C is further adjusted for linguistic distance  ^4^ Model D is further adjusted for discrimination | | | | | |
| Odds ratios in **bold** are statistically significant (p<0.05) | | | | | |

*Secondary outcomes*

Supplemental Tables 7 and 8 detail the results by dichotomised diagnostic category. Broad patterns for non-affective psychotic disorders (n=2,256, Supplemental Table 7) were similar to the results from all psychotic disorders. Crude excess odds were observed in all ethnic minority groups (OR: 2.08, 95%CI: 1.70-2.57) and were highest in the North-African (OR: 7.82, 95%CI: 2.73-853) and the black (OR: 2.68, 95%CI: 1.97-3.66) groups. Following adjustment for confounders (model A), excess risk remained (OR for all ethnic minorities 1.64, 95%CI: 1.29). Following further adjustment for social disadvantage (Model B) the overall excess risk remained similar (OR: 1.59, 95%CI1.23-2.04), but this masked heterogeneity between ethnic minority group. Second- and later-generation minorities were no longer at significantly increased risk (OR: 1.18, 95%CI:0.84-1.66). When linguistic distance was added to the Model (Model C), only the North-African group retained excess odds (OR: 2.13, 95%CI: 1.01-4.51). In the final model (D, including discrimination), this was no longer the case.

Patterns were again similar, though not as strong, for affective disorders (n=1,801, Supplemental Table 8). Crude excess odds were observed in the overall ethnic minority group (OR: 1.84, 95%C: 1.40-2.42) and in the black (OR:2.05, 95%CI:1.37-3.09) and mixed (OR:2.61, 95%CI: 1.72-3.98) groups. First-generation groups no longer face excess odds following adjustment for confounders (Model A), and second-generation minorities and the black minority group no longer faced excess odds after adjustment for social disadvantage (Model B). Following further adjustment for linguistic distance (Model C), 95% confidence intervals overlapped with the null for all minority group, and further adjusting for discrimination (Model D) did not alter odds ratios. The results for affective disorders have to be interpreted with a degree of caution: due to the small (n=306) number of cases there is a lack of precision surrounding the point estimates.

**Supplemental Table 7: Odds of non-affective psychotic disorder by exposure status following incremental covariate adjustment (n=2,256)**

| Model | Crude | Model A^1^ | Model B^2^ | Model C^3^ | Model D^4^ |
| --- | --- | --- | --- | --- | --- |
| Variable | OR (95%CI) | OR (95%CI) | OR (95%CI) | OR (95%CI) | OR (95%CI) |
| Ethnicity  White native  All minorities  Black  Mixed  Asian  North-African  Other  White other | Reference  **2.08 (1.70-2.57)**  **2.68 (1.97-3.66)**  **2.07 (1.43-2.99)**  **1.85 (1.06-3.22)**  **7.82 (2.73-8.53)**  **1.93 (1.06-3.55)**  1.30 (0.91-1.87) | Reference  **1.64 (1.29-2.07)**  **1.89 (1.33-2.69)**  **1.55 (1.02-2.38)**  1.71 (0.93-3.15)  **4.22 (2.21-8.06)**  1.50 (0.77-2.92)  1.17 (0.78-1.74) | Reference  **1.59 (1.23-2.04)**  **1.62 (1.11-2.37)**  1.15 (0.72-1.81)  **2.28 (1.16-4.8)**  **3.51 (1.76-7.00)**  1.79 (0.88-3.64)  1.45 (0.9-2.24) | Reference  1.17 (0.87-1.58)  1.38 (0.93-2.03)  1.08 (0.68-1.70)  1.56 (0.76-3.20)  **2.13 (1.01-4.51)**  1.24 (0.59-2.65)  0.94 (0.67-1.54) | Reference  1.14 (0.95-1.54)  1.33 (0.90-1.98)  1.06 (0.67-1.68)  1.45 (0.75-3.16)  2.07 (0.98-4.39)  1.06 (0.7-2.59)  0.93 (0.56-1.53) |
| Generational status  First generation  Later generations | **2.00 (1.57-2.56)**  **2.20 (1.67-2.90)** | **1.76 (1.33-2.32)**  **1.50 (1.10-2.05)** | **2.01 (1.48-2.71)**  1.18 (0.84-1.66) | 1.32 (0.88-1.97)  1.13 (0.80-1.59) | 1.29 (0.86-1.93)  1.10 (0.77-1.56) |
| Parental SES  Professional  Intermediate  Lower  Routine  Never worked  Not classified | Reference  **1.33 (1.01-1.76)**  **1.63 (1.26-2.12)**  **1.95 (1.44-2.64)**  **11.39 (3.18-40.69)**  **2.89 (1.83-4.55)** | n/a | Reference  1.14 (0.82-1.58)  1.23 (0.89-1.70)  1.05 (0.73-1.53)  **3.93 (1.00-15.44)**  **2.61 (1.53-4.45)** | Reference  1.15 (0.83-1.60)  1.25 (0.90-1.73)  1.09 (0.76-1.59)  3.86 (0.98-15.29)  **2.60 (1.52-4.43)** | Reference  1.14 (0.82-1.60)  1.24 (0.90-1.72)  1.09 (0.76-1.58)  3.86 (0.97-15.28)  **2.59 (1.51-4.42)** |
| Level of education  Postgraduate  Undergraduate  Vocational  Tertiary  School qualifications  No qualifications | Reference  **1.60 (1.04-2.49)**  **3.95 (2.58-6.03)**  **2.86 (1.88-4.34)**  **7.54 (4.93-11.53)**  **15.27 (9.36-24.89)** | n/a | Reference  1.52 (0.94-2.45)  **2.85 (1.77-4.57)**  **1.70 (1.07-2.71)**  **5.00 (3.11-8.06)**  **8.83 (5.03-15.48)** | Reference  1.53 (0.95-2.48)  **2.91(1.80-4.69)**  **1.72 (1.08-2.75)**  **5.10 (3.16-8.24)**  **8.87 (5.05-15.59)** | Reference  1.54 (0.95-2.49)  **2.90 (1.80-4.67)**  **1.73 (1.08-2.74)**  **5.12 (3.17-8.26)**  **8.92 (5.08-15.67)** |
| Relationship status  No  Yes | Reference  **0.22 (0.17-0.27)** | n/a | Reference  **0.28 (0.21-0.37)** | Reference  **0.28 (0.21-0.37)** | Reference  **0.28 (0.21-0.40)** |
| Living arrangements  No  Yes | Reference  **0.41 (0.33-0.50)** | n/a | Reference  0.79 (0.60-1.06) | Reference  0.77 (0.58-1.03) | Reference  0.77 (0.58-1.03) |
| Linguistic distance  No  Yes | Reference  **2.08 (1.60-2.71)** | n/a | n/a | Reference  **2.11 (1.40-3.18)** | Reference  **2.10 (1.39-3.17)** |
| Discrimination (0-12) | **1.21 (1.12-1.31)** | n/a | n/a | n/a | 1.05 (0.95-1.16) |
| ^1^ Model A is adjusted for covariates (age, sex, their interaction, paternal age, childhood trauma, cannabis use, parental history of psychosis)  ^2^ Model B is further adjusted for indicators of social disadvantage (paternal SES, level of education, relationship status and living arrangements)  ^3^ Model C is further adjusted for linguistic distance  ^4^ Model D is further adjusted for discrimination | | | | | |
| Odds ratios in **bold** are statistically significant (p<0.05) | | | | | |

**Supplemental Table 8: Odds of affective psychotic disorders by exposure status following incremental covariate adjustment (n=1,801)**

| Model | Crude | Model A^1^ | Model B^2^ | Model C^3^ | Model D^4^ |
| --- | --- | --- | --- | --- | --- |
| Variable | OR (95%CI) | OR (95%CI) | OR (95%CI) | OR (95%CI) | OR (95%CI) |
| Ethnicity  White native  All minorities  Black  Mixed  Asian  North-African  Other  White other | Reference  **1.84 (1.40-2.42)**  **2.05 (1.37-3.09)**  **2.61 (1.72-3.98)**  1.70 (0.79-3.66)  1.90 (0.77-4.72)  1.41 (0.56-3.55)  0.86 (0.47-1.59) | Reference  **1.46 (1.09-1.98)**  **1.56 (1.00-2.43)**  **1.93 (1.22-3.06)**  1.90 (0.86-4.18)  1.52 (0.58-3.92)  0.86 (0.32-2.30)  0.82 (0.43-1.55) | Reference  **1.38 (1.01-1.89)**  1.31 (0.83-2.09)  **1.64 (1.00-2.67)**  **2.56 (1.13-5.83)**  1.17 (0.42-3.30)  1.07 (0.38-2.95)  0.92 (0.47-1.78) | Reference  1.22 (0.85-1.74)  1.16 (0.72-1.87)  1.57 (0.96-2.56)  1.84 (0.76-4.46)  0.78 (0.26-2.37)  0.75 (0.25-2.22)  0.65 (0.30-1.35) | Reference  1.19 (0.83-1.71)  1.12 (0.69-1.81)  1.54 (0.94-2.53)  1.81 (0.75-4.41)  0.77 (0.25-2.34)  0.69 (0.23-2.08)  0.65 (0.31-1.36) |
| Generational status  First generation  Later generations | **1.63 (1.13-2.35)**  **1.98 (1.40-2.79)** | 1.40 (0.94-2.08)  **1.46 (1.01-2.12)** | 1.48 (0.98-2.24)  1.24 (0.83-1.84) | 1.11 (0.63-1.95)  1.22 (0.82-1.82) | 1.08 (0.62-1.91)  1.20 (0.80-1.78) |
| Parental SES  Professional  Intermediate  Lower  Routine  Never worked  Not classified | Reference  1.16 (0.79-1.72)  1.28 (0.88-1.85)  1.38 (0.90-2.10)  4.41 (0.70-27.91)  1.61 (0.85-3.08) | n/a | Reference  0.96 (0.63-1.46)  0.89 (0.59-1.34)  0.71 (0.44-1.14)  1.74 (0.23-12.96)  1.41 (0.69-2.84) | Reference  0.97 (0.64-1.49)  0.90 (0.60-1.37)  0.74 (0.46-1.21)  1.94 (0.25-15.24)  1.44 (0.71-2.90) | Reference  0.97 (0.63-1.48)  0.90 (0.59-1.36)  0.74 (0.45-1.19)  1.91 (0.24-15.31)  1.44 (0.71-2.91) |
| Level of education  Postgraduate  Undergraduate  Vocational  Tertiary  School qualifications  No qualifications | Reference  **1.73 (0.87-3.43)**  **3.55 (1.81-6.99)**  **3.80 (2.00-7.22)**  **8.01 (4.17-15.35)**  **12.36 (6.09-25.12)** | n/a | Reference  1.68 (0.83-3.37)  **3.00 (1.48-6.07)**  **3.12 (1.60-6.07)**  **6.61 (3.33-13.09)**  **9.58 (4.47-20.52)** | Reference  1.68 (0.83-3.38)  **2.93 (1.44-5.95)**  **3.11 (1.60-6.07)**  **6.53 (3.30-12.97)**  **9.23 (4.30-19.81)** | Reference  1.71 (0.85-3.44)  **2.94 (1.45-5.97)**  **3.14 (1.61-6.12)**  **6.61 (3.33-13.12)**  **9.44 (4.40-20.23)** |
| Relationship status  No  Yes | Reference  **0.42 (0.30-0.58)** | n/a | Reference  **0.52 (0.34-0.76)** | Reference  **0.52 (0.35-0.77)** | Reference  **0.52 (0.35-0.76)** |
| Living arrangements  No  Yes | Reference  **0.68 (0.50-0.93)** | n/a | Reference  0.94 (0.63-1.38) | Reference  0.90 (0.61-1.33) | Reference  0.90 (0.61-1.33) |
| Linguistic distance  No  Yes | Reference  **1.71 (1.16-2.53)** | n/a | n/a | Reference  **1.87 (1.07-3.23)** | Reference  **1.84 (1.07-3.18)** |
| Discrimination (0-12) | **1.26 (1.13-1.42)** | n/a | n/a | n/a | 1.09 (0.95-1.25) |
| ^1^ Model A is adjusted for covariates (age, sex, their interaction, paternal age, childhood trauma, cannabis use, parental history of psychosis)  ^2^ Model B is further adjusted for indicators of social disadvantage (paternal SES, level of education, relationship status and living arrangements)  ^3^ Model C is further adjusted for linguistic distance  ^4^ Model D is further adjusted for discrimination | | | | | |
| Odds ratios in **bold** are statistically significant (p<0.05) | | | | | |

*Sensitivity analyses*

Supplemental Table 9 details the results of sensitivity analyses, where all main analyses were repeated on the complete case sample using inverse probability weights. Broad patterns were similar to the imputed data, increasing confidence in the imputation model. In the crude model, there was no loss due to missing data. By the final model, only 66.7% (n=1,722) of observations remained.

**Supplemental Table 9: Sensitivity analyses of the associations between FEP risk and ethnic group, by statistical model (complete cases only, using inverse probability weights)**

|  | **Crude** | **Model A^1^** | **Model B^2^** | **Model C^3^** | **Model D^4^** |
| --- | --- | --- | --- | --- | --- |
| *N (%)* | *2,583 (100.0)* | *2,068 (80.0)* | *1995 (77.2%)* | *1995 (77.2%)* | *1722 (66.7%)* |
|  | OR (95%CI) | OR (95%CI) | OR (95%CI) | OR (95%CI) | OR (95%CI) |
| **Ethnicity**  White majority  All minorities  Black  Mixed  Asian  North-African  Other  White other | Reference  **1.96 (1.70-2.27)**  **2.31 (1.71-3.12)**  **2.28 (1.52-3.42)**  1.38 (0.77-2.46)  **4.39 (1.74-11.03)**  **1.91 (0.74-4.97)**  1.20 (0.84-1.71) | Reference  **1.41 (1.13-1.75)**  **1.56 (1.14-2.12)**  **1.58 (1.04-2.40)**  1.06 (0.53-2.14)  **3.67 (1.79-7.49)**  1.36 (0.51-3.56)  0.87 (0.57-1.34) | Reference  **1.31 (1.08-1.59)**  1.27 (0.87-1.86)  1.25 (0.85-1.85)  1.38 (0.72-2.66)  **3.14 (1.51-6.54)**  1.35 (0.53-3.41)  0.99 (0.65-1.52) | Reference  1.05 (0.84-1.30)  1.10 (0.76-1.58)  1.19 (0.79-1.80)  0.94 (0.55-1.62)  1.86 (0.84-4.10)  0.99 (0.38-2.59)  0.65 (0.40-1.06) | Reference  1.06 (0.85-1.32)  1.11 (0.75-1.64)  1.20 (0.76-1.91)  0.72 (0.45-1.15)  1.88 (0.80-4.43)  1.25 (0.42-3.76)  0.67 (0.42-1.1.09) |
| **Generation**  First  Later | **1.90 (1.62-2.24)**  **1.97 (1.62-2.41)** | **1.55 (1.20-1.91)**  1.29 (0.97-1.72) | **1.60 (1.33-1.92)**  1.05 (0.85-1.31) | 1.09 (0.82-1.45)  1.01 (0.80-1.29) | 1.08 (0.80-1.46)  1.03 (0.80-1.32) |
| ^1^: Model A is adjusted for covariates (age, sex paternal age, childhood trauma, cannabis use and parental history of psychosis)  ^2^: Model B is further adjusted for social disadvantage (paternal socioeconomic status, level of education, relationship status, living arrangements)  ^3^: Model C is further adjusted for linguistic distance  ^4^: Model D is further adjusted for discrimination | | | | | |
| Odds ratios in **bold** are significant (p<0.05) | | | | | |

**Supplemental bibliography**

European Network of National Schizophrenia Networks Studying Gene-Environment Interactions. (2013). *EU-GEI study protocol (Cambridgeshire and Peterborough)* (Vol. 31, pp. 1–22). Vol. 31, pp. 1–22.

Jongsma, H. E., Gayer-Anderson, C., Lasalvia, A., Quattrone, D., Mulè, A., Szöke, A., … Kirkbride, J. B. (2018). Treated Incidence of Psychotic Disorders in the Multinational EU-GEI Study. *JAMA Psychiatry*, *75*(1), 36. https://doi.org/10.1001/jamapsychiatry.2017.3554

Sullivan, S. A., Hollen, L., Wren, Y., Thompson, A. D., Lewis, G., & Zammit, S. (2016). A longitudinal investigation of childhood communication ability and adolescent psychotic experiences in a community sample. *Schizophrenia Research*, *173*(1–2), 54–61. https://doi.org/10.1016/j.schres.2016.03.005

1. NB: this is not a main publishable output, as this will be covered by Jean-Paul Selten and colleagues in more detail, but must be included here to allow testing of subsequent hypotheses. [↑](#footnote-ref-1)
